# Polar Nitride Perovskite LaWN_3‐*δ*_ with Orthorhombic Structure

**DOI:** 10.1002/advs.202205479

**Published:** 2023-05-02

**Authors:** Xuefeng Zhou, Wenwen Xu, Zhigang Gui, Chao Gu, Jian Chen, Jianyu Xie, Xiaodong Yao, Junfeng Dai, Jinlong Zhu, Liusuo Wu, Er‐jia Guo, Xiaohui Yu, Leiming Fang, Yusheng Zhao, Li Huang, Shanmin Wang

**Affiliations:** ^1^ Department of Physics & Academy for Advanced Interdisciplinary Studies Southern University of Science & Technology Shenzhen Guangdong 518055 China; ^2^ Quantum Science Center of Guangdong‐Hongkong‐Macao Greater Bay Area Shenzhen Guangdong 518055 China; ^3^ Beijing National Laboratory for Condensed Matter Physics and Institute of Physics Chinese Academy of Sciences Beijing 100190 China; ^4^ Key Laboratory for Neutron Physics Institute of Nuclear Physics and Chemistry China Academy of Engineering Physics Mianyang 621999 China

**Keywords:** high‐pressure synthesis, LaWN_3_, nitride perovskites, polar structure

## Abstract

Nitride perovskite LaWN_3_ has been predicted to be a promising ferroelectric material with unique properties for diverse applications. However, due to the challenging sample preparation at ambient pressure, the crystal structure of this nitride remains unsolved, which results in many ambiguities in its properties. Here, the authors report a comprehensive study of LaWN_3_ based on high‐quality samples synthesized by a high‐pressure method, leading to a definitive resolution of its crystal structure involving nitrogen deficiency. Combined with theoretical calculations, these results show that LaWN_3_ adopts an orthorhombic *Pna2*_1_ structure with a polar symmetry, possessing a unique atomic polarization along the *c‐*axis. The associated atomic polar distortions in LaWN_3_ are driven by covalent hybridization of W: 5d and N: 2p orbitals, opening a direct bandgap that explains its semiconducting behaviors. The structural stability and electronic properties of this nitride are also revealed to be closely associated with its nitrogen deficiency. The success in unraveling the structural and electronic ambiguities of LaWN_3_ would provide important insights into the structures and properties of the family of nitride perovskites.

## Graphical abstract

High‐quality nitride perovskite LaWN_3‐*δ*_ has been successfully synthesized using a high‐pressure reaction route, leading to a definitively identified structure of orthorhombic *Pna2*_1_‐LaWN_3‐*δ*_ with a polar symmetry. The nitrogen deficiency and hybridization of W:*t*_2g_ and N:p states are found to be crucial for affecting its structural stability and electronic properties, offering powerful insights towards understanding the properties of nitride perovskites.

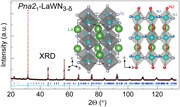

## 1 Introduction

Ternary metal nitrides have recently reemerged as an exciting frontier for materials study,^[^ ^1^, ^2^, ^3^ ^]^ because there exist a large number of unexplored metastable functional nitrides awaiting experimental discovery.^[^ ^4^, ^5^, ^6^, ^7^, ^8^, ^9^ ^]^ Of particular interest is the nitride with a perovskite structure of ABN_3_, where cations A and B are limited to a narrow domain of elements due to a high valence state of nitrogen.^[^ ^6^, ^10^ ^]^ Both the structures and compositions of nitride perovskites are expected to be largely tunable for producing exceptional diversity of nitrides. Among them, LaWN_3_ is the most attractive nitride with a predicted polar symmetry, showing promising switchable ferroelectricity and robust polarization.^[^ ^6^, ^11^ ^]^ These, combined with excellent mechanical stiffness and ease of integration with semiconductors, make it technologically relevant for nonvolatile memories, acoustic resonators, and more.^[^ ^8^, ^12^, ^13^ ^]^ However, the crystal structures of most nitride perovskites including LaWN_3_ still remain elusive,^[^ ^6^, ^8^, ^10^, ^14^, ^15^, ^16^, ^17^, ^18^, ^19^, ^20^, ^21^ ^]^ which have led to conflicting predictions (e.g., *R*3*c* ^[^ ^6^, ^7^, ^10^, ^11^, ^19^, ^20^, ^21^ ^]^ and *Pna2*_1_^[^ ^17^, ^20^ ^]^) and imposed an insurmountable barrier for exploring this important class of materials.

The difficulty in definitive structural resolution of nitride perovskites is primarily due to the challenging sample preparation using traditional approaches at ambient pressure.^[^ ^1^, ^4^, ^5^ ^]^ In fact, most of those nitrides are thermodynamically unstable at atmospheric pressure and readily decompose by degassing N_2_.^[^ ^1^, ^4^ ^]^ Besides, oxygen is usually involved and difficult to avoid as an impurity species in preparing oxygen‐free nitrides. As a result, most previously reported products are poorly‐crystallized oxynitrides (e.g., LaWO_0.6_N_2.4_) with unwanted impurities,^[^ ^22^, ^23^, ^24^, ^25^, ^26^ ^]^ hindering solving their structures. An oxygen‐free thin‐film LaWN_3_ sample has recently been prepared; the piezoelectric measurement suggests it has a polar structure.^[^ ^13^, ^27^ ^]^ However, the crystallinity of the thin‐film sample is still insufficient to produce excellent X‐ray and electron diffraction signals for achieving definitive structural resolution, and there exists a number of structures that cannot be discernible including polar symmetries of *R3c*, *P4mm*, *Pmc2*_1_, and *Pna2*_1_. Because the polar structure is closely associated with the N atoms that are insensitive to both the x‐ray and electrons, it is difficult to determine ferroelectric distortions by conventional methods.

The methodological advancement towards synthesizing high‐quality LaWN_3_ samples is crucial for addressing the associated issues. High pressure (*P*) and temperature (*T*) synthesis is in this regard a powerful approach for preparation of nitrogen‐rich metal nitrides.^[^ ^28^, ^29^ ^]^ A surge of recent studies along this direction has led to the discovery of many exotic metal nitrides with promising properties.^[^ ^28^, ^29^, ^30^, ^31^, ^32^, ^33^ ^]^ However, most of those syntheses involve a direct nitridation of metal and the required pressure is high (i.e., above 10 GPa), which is beyond the current technological capability for massive production. Using soft solid‐state reaction routes, numerous nitrogen‐rich nitrides have been obtained at moderate pressures.^[^ ^9^, ^34^, ^35^, ^36^, ^37^ ^]^ In stark contrast, reports on high‐P synthesis of nitride perovskites are sparse except for a recently‐reported triclinic LaReN_3_ metal.^[^ ^38^, ^39^ ^]^

Here, we extend the high P‐T synthesis to the family of nitride perovskites with a focus on LaWN_3_ by means of soft reaction routes and successfully synthesize high‐quality LaWN_3_ samples. The definitive structure of this nitride is determined to have a polar *Pna*2_1_ symmetry, rather than previously proposed *R*3*c* based on thin‐film samples.^[^ ^27^ ^]^ The stabilities and properties of these samples are then explored, which provide powerful insights into the electronic origin of structural stability and the structure‐property relationship.

## 2 Results and Discussion

A favorable route is exploited to synthesize LaWN_3_ from a reaction between La_2_W_2_O_9_ and NaNH_2_ at 2–5 GPa and temperatures of 600–2100 °C, leading to high‐quality nitride samples. The material is compositionally constituted by La, W, and N with a molar ratio of ≈1:1:3 as determined by energy‐dispersive X‐ray (EDX) experiments (Figures S1–S4, Supporting Information). Note that the nitride can also be synthesized from a distinct reaction between LaN, W, and NaNH_2_ without involvement of oxygen (Figure S3, Supporting Information), suggesting it is oxygen‐free LaWN_3_. Complicating matter further is that our AES measurements indicate a small amount of oxygen may be involved, giving rise to a possible composition of LaWO*_x_* N_3‐_ *_x_* (*x* ≈ 0.25) (Figure S4, Supporting Information). However, for most transition‐metal (TM) nitrides, the oxygen concentration cannot be accurately determined solely by any one of the commonly accessible methods. Instead, it requires complementary experimental measurements including AES, EDX, XPS, TG‐MS, and structural refinements using XRD and NPD data, combined with careful analysis. Bearing this in mind, we have determined the oxygen concentration in our sample, showing a nearly negligible level with *x* ≈ 0 in LaWO*_x_* N_3‐_ *_x_*, and more details will be shown below. Because the sample can be prepared at a moderate pressure of 2–5 GPa, the lowest among high‐P syntheses of ternary metal nitrides, it is practically feasible for massive and industrial‐scale production.

**Figure 1:**
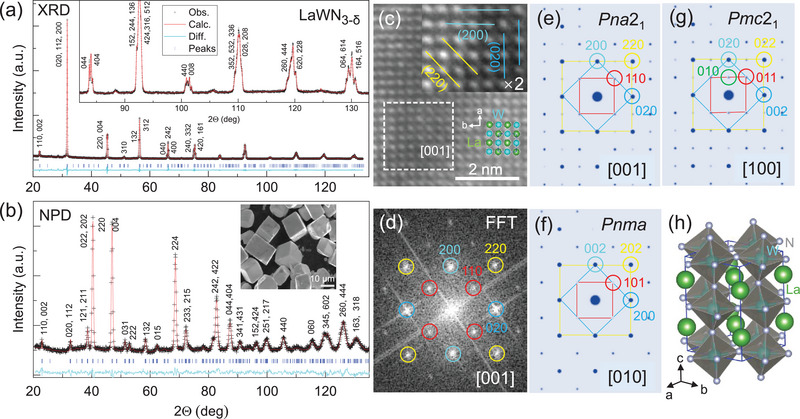
Crystal structure of LaWN_3‐*δ*_. a–b) Refined ambient XRD and NPD patterns. c) TEM image with an enlargement to show the (010), (100), and (110) fringes. d) SED pattern obtained by an FFT of the selected area in (c). e–g) Simulated SED patterns using the refined *Pna*2_1_, *Pnma*, and *Pmc*2_1_, respectively. (h) Refined *Pna*2_1_‐LaWN_3‐*δ*_.

All the observed X‐ray diffraction (XRD) peaks of the sample can initially be indexed by a $Pm\bar{3}m$‐perovskite structure with *a* ≈ 3.992 Å (**Figure** **1**a), especially for the samples synthesized below 1200 °C, showing increasing peak broadening as the temperature decreases (Figure S1, Supporting Information). This, combined with the disordered lattice (Figure S2, Supporting Information), indicates a low sample crystallinity. However, for the samples synthesized above 1600 °C, the single‐crystal samples can be obtained with crystallite size up to 200 µm (Figure S1, Supporting Information), and each the otherwise broadened peak of the low‐T sample splits into a set of reflection lines (Figure 1a), indicative of a low‐symmetry derivative of cubic perovskite. Thus, the previously proposed rhombohedral and tetragonal structures are quickly excluded such as *R*3*c* and *I‐4* $\bar{4}$ ^[^ ^19^, ^25^, ^27^ ^]^ (Figures S5‐S6, Supporting Information). Instead, the XRD pattern can only be appropriately indexed with an orthorhombic cell. Due to the insensitivity of N to X‐rays, the structure of LaWN_3_ cannot be determined by the X‐ray‐only experiment. Indeed, several symmetries can reach the same refinement including *Pnma*, *Pna*2_1_, and *Pmc*2_1_ (Figure S7, Supporting Information), making them indistinguishable.

We thus perform the neutron powder diffraction (NPD) measurements to accurately determine nitrogen, because N has a greater scattering length than those of La and W.^[^ ^40^ ^]^ The resultant NPD pattern in Figure 1b has a different subset of strong peaks that are almost invisible in the XRD pattern. Although the different N positions can be well refined for each orthorhombic model using our NPD data, a similar excellence of refinement is achieved and hence the structural ambiguity still retains. Using the refined *Pnma*, *Pna*2_1_, and *Pmc*2_1_, we simulate their electron powder diffraction (EPD) patterns, showing apparent peak differences in the low‐2Θ range mainly associated with the N atoms (Figures S8‐S9, Supporting Information). This allows identifying the structure of LaWN_3‐*δ*_ by analyzing single‐crystal electron diffraction (SED) along the [001] direction (Figure 1c).

A fast Fourier transformation (FFT) of the selected area of transmission electron microscopy (TEM) image gives a characteristic 110 diffraction spot (Figures 1c–d)), consistent with the simulated using *Pna*2_1_ (Figure 1e), whereas other models cannot correctly reproduce the observed diffraction pattern (Figures 1f–g). We thus conclude that the polar *Pna2*_1_ symmetry is most suitable for LaWN_3‐*δ*_. Note that the polar symmetry is also strongly evidenced by our SHG experiments (Figure S10, Supporting Information). The refined lattice parameters are summarized in **Table** **1** and the crystal structure is plotted in Figure 1h and Figure S11, Supporting Information. The presence of vacancies at N1 and N2 sites leads to a composition of LaWN_2.6_. Note that previous calculations by Fang et al. indicate that stoichiometric *Pna*2_1_‐LaWN_3_ is a metastable phase, although it is dynamically favorable.^[^ ^11^ ^]^ The presence of atomic defects may make *Pna*2_1_ thermodynamically more stable, rather than the predicted ground state of *R*3*c*. However, for the previous thin‐film samples prepared at a relatively low temperature of 900 °C by Talley et al.,^[^ ^27^ ^]^ the sample crystallinity is seemingly not enough for obtaining high‐quality XRD data with sharp diffraction peaks, especially for the high‐angle peaks, similar to that of our high‐P samples synthesized at 5 GPa and below 1200 °C (see Figure S1, Supporting Information). Clearly, using XRD patterns with broadened peak profiles, it is difficult to determine its definitive crystal structure, leading to a misassigned *R*3c for LaWN_3‐*δ*_. From this viewpoint, the crystal structure of such‐reported thin‐film samples should also be *Pna*2_1_, rather than *R*3c.

**Table 1:** Refined lattice parameters for *Pna*2_1_‐LaWN_3‐*δ*_

	Orthorhombic, *Pna*2_1_‐LaWN_3‐*δ*_ at ambient conditions
Cell parameters	*a* = 5.6384 (2) Å, *b* = 5.6643 (2) Å, *c* = 7.9614 (3) Å
Symmetry	*Pna*2_1_ (No. 33)
Volume, density	254.27 Å^3^, 9.529 g cm^−3^

Atomic position	Wyckoff site	Occupancy
	La: 4*a* (0, 0, z) (z ≈ 1/4) (XRD)*	1
	W: 4*a* (x, 0, 0) (x ≈ 1/2) (XRD)*	1
	N1: 4*a* (0.243, 0.248, 0.536) (NPD)	0.72
	N2: 4*a* (0.481, 0.044, 0.245) (NPD)	0.88
	N3: 4*a* (0.238, 0.764, 0.487) (NPD)	1
wRp (%)	5.3 for XRD; 4.7 for NPD	

* The refined *z_La_* = 0.252 (2) and *x_W_* = 0.499 (1) are almost identical to their ideal coordinates of ¼ and ½, respectively, by which a same excellence of refinement reaches.

**Figure 2:**
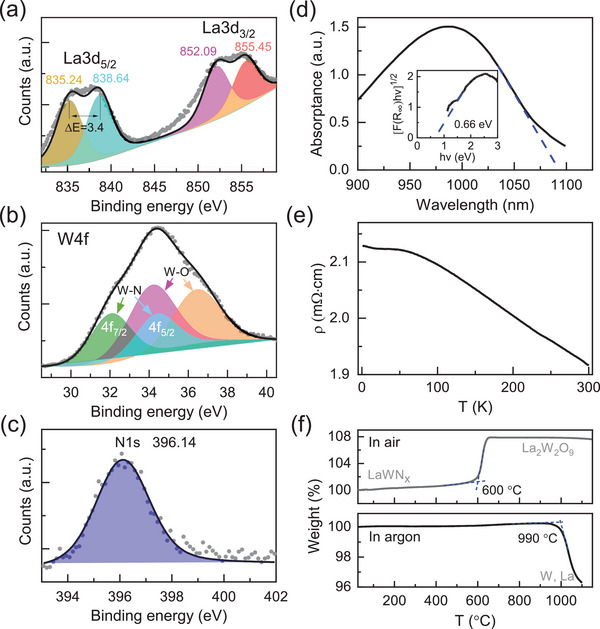
Electronic structure and thermal stability. a–c) XPS spectra. d) UV‐Vis absorption spectrum with a limited wavelength coverage of 300 – 1100 nm. The absorption edge is denoted by dashed line. Inset is a Tauc's plot for determining bandgap. e) Low‐T resistivity measurement. f) Thermal stability measurements.

To explore the electronic structure of LaWN_3‐*δ*_, the binding energies of La3d, W4f, and N1s are determined by x‐ray photoelectron spectroscopy (XPS) measurements. Due to the spin‐orbit coupling, both the La3d_5/2_ and 3d_3/2_ states split into a doublet with a similar energy split of ∆*E* ≈ 3.4 eV (**Figure** **2**a), close to that of nearly purely ionic La^3+[^ ^41^, ^42^ ^]^ (Figure S13, Supporting Information), indicating that La has an excellent electropositivity in analogy to alkali and alkaline‐earth metals for donating electrons during reaction. Thus, the role of La played in the formation LaWN_3‐*δ*_ is to donate electrons to its adjacent N, leading to nitrogen reduction; this in turn oxidizes more electronegative W and eventually drives covalent hybridization of W and N under the inductive effect.^[^ ^4^ ^]^ In fact, the determined high hardness of the sample (i.e., ≈9 GPa) also suggests its strong covalency (Figure S12, Supporting Information). In Figure 2b, the doublet of W4f_7/2_ and 4f_5/2_ for LaWN_2.6_ has intermediate binding energies between those of W and WO_2_,^[^ ^43^ ^]^ using the established relationship between the binding energy and valence state of W, based on a number of known W‐bearing materials (see Figure S13, Supporting Information). The thus‐evaluated valence W^2+^ is much distinct from the nominal valence W^4.8+^, further signaling the strong W‐N covalency. Due to the surface oxidation effect, an additional W4f doublet is also observed and likely associated with a WO_2+_ *_x_* compound,^[^ ^44^ ^]^ which can profoundly be reduced if the sample surface is etched and cleaned by Ar^+^ irradiation before experiments (Figure 2b and Figure S4j), Supporting Information. By contrast, the La3d line are nearly intact after etching [Figure S4(k)], implying that LaWN_3‐*δ*_ has a similar valence state to that of the surface La‐O oxide; this is not unexpected because La is so electropositive for donating the same number of electrons during the formation of LaWN_3‐*δ*_ or surface La–O oxide. For the N1s, a small binding energy is determined with a value close to that of ScN semiconductor (i.e., ≈396.1 eV) (Figure 2c),^[^ ^45^ ^]^ implying a similar metal‐N bonding state for opening a bandgap. Our ultraviolet‐visible (UV‐Vis) absorption experiment of the sample shows a clear absorption edge with a small bandgap of ≈0.66 eV, in conjunction with resistivity measurements (Figures 2d–e), suggesting it is likely a semiconductor. Besides, its resistivity is ≈2 mΩ cm, comparable with that of ScN with a bandgap of ≈0.9 eV^[^ ^46^ ^]^ or graphite with characteristics of a bad metal. Such an unexpectedly small bandgap in LaWN_2.6_ is probably due to the suppression of polar distortion that results from nitrogen deficiency as discussed later (Figure S18, Supporting Information). Besides, due to the electron‐filling effect induced by nitrogen deficiency, our calculations show that LaWN_2.6_ should have metallic band structures (Figure S18, Supporting Information), inconsistent with semiconducting behaviors as observed in optical absorption experiments. Such contraindicated properties may be attributed to the size effect and other types of crystalline imperfections such as lattice disorder/stress, atomic deficiencies at cation positions, and low crystallinity, which would greatly alter electronic properties of nitride materials, as previously reported in other metal nitrides (e.g., CrN and TiN).^[^ ^47^, ^48^, ^49^, ^50^ ^]^ Those intriguing properties of metal nitrides are probably attributed to the unique electronegativity of N.^[^ ^4^, ^5^ ^]^

Our thermogravimetric analysis indicates LaWN_2.6_ starts to oxidize at ≈600 °C in air and transforms into La_2_W_2_O_9_ (Figure 2f), by which the nitrogen concentration *x* in LaWN*_x_* is estimated to be ≈2.5(5), consistent with the refined *x* = 2.6. In argon, it decomposes by degassing N_2_ at ≈990 °C. The situation is greatly changed at high pressure, and the sample's thermal stability is profoundly prompted up to 2100 °C at 5 GPa without obvious decomposition (Figure S3, Supporting Information), showing the effectiveness of pressure for synthesizing nitrides. Nevertheless, the nitrogen deficiency of the high‐P samples is still difficult to alleviate, mainly because of a relatively smaller electronegativity of nitrogen, compared with oxygen.

**Figure 3:**
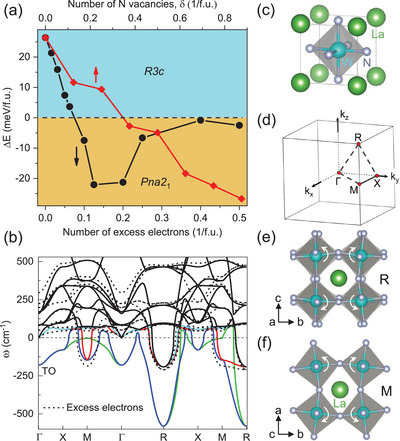
Phase stability and phonon dispersion. a) Energy difference, $\Delta E\;=\;E_{Pna2_{1}}-E_{R3c}$, versus electron doping and nitrogen vacancies, *δ*, in LaWN_3‐*δ*_. b) Phonon dispersion of pristine$Pm\bar{3}m$‐LaWN_3_ (solid lines) and the case with electron doping. There are 0.375 electrons added into the conduction band (dashed lines). (c)‐(d) Structure and Brillouin zone of cubic $Pm\bar{3}m$‐LaWN_3_. e–f) Rotation of WN_6_ octahedra associated with unstable R and M models.

To examine the dependence of phase stability on nitrogen deficiency, the energies of two competing structures of *R*3*c* and *Pna*2_1_ are calculated by introducing nitrogen vacancies (*δ*) in LaWN_3‐*δ*_.^[^ ^51^, ^52^, ^53^, ^54^, ^55^, ^56^, ^57^ ^]^ In **Figure** **3**a, *R*3*c* is more stable if *δ* is below 0.3, while increasing *δ* above 0.375 can lead to a *R*3*c*‐to‐*Pna*2_1_ crossover, which well explains the reason why *Pna*2_1_‐LaWN_2.6_ is more favorable for our samples. In principle, the nitrogen vacancies serve as electron donors and make the material conductive. To check this, we performed calculations by adding excess electrons into its conduction band, resulting in a similar trend of stabilities for *R*3*c* and *Pna*2_1_. The most stable *Pna*2_1_‐ LaWN_3‐*δ*_ can be achieved when an additional 0.124 – 0.20 electrons/formula cell (f.u.) are added, indicating that nitrogen deficiency is key to stabilizing *Pna*2_1_‐LaWN_2.6_. On the other hand, the thus‐enhanced metallicity of LaWN_2.6_ with free carries would screen the long‐range Coulomb field^[^ ^58^ ^]^ and hence destabilize ferroelectricity (see Figures S17‐S18, Supporting Information).

Exploring the lattice instabilities of cubic‐LaWN_3_ allows for gaining deep insights for rationalizing the stability of *Pna*2_1_‐LaWN_2.6_. Phonon dispersions of cubic‐LaWN_3_ along the high‐symmetry lines of ГX, ГM, and ГR correspond to the [100], [110], and [111] directions of the cell,^[^ ^57^ ^]^ respectively (Figure 3c–d). It can be seen that the imaginary frequencies mainly arise from a branch of transverse optic (TO) unstable modes that are primarily associated with the W and N atoms.

The unstable R and M modes are related to antiphase and in‐phase antiferrodistortive instabilities, characterized by the rotation of neighboring WN_6_ octahedra (Figure 3e–f), as commonly seen in oxide perovskites like SrTiO_3_.^[^ ^59^ ^]^ The unstable modes near Г are dominated by a relative W‐N displacement, which breaks the centrosymmetric symmetry, similar to that in BaTiO_3_.^[^ ^60^ ^]^ The phonon instabilities along the Г‐X line can be alleviated and even quenched if enough free electrons are added in Figure 3b), as indicated above. Meanwhile, the instability at *R* is substantially reduced, which is in contrast to that at *M* with increased instability, indicating the orthorhombic symmetry becomes more favorable. Therefore, such unique crystal and electronic properties of our LaWN_3‐*δ*_ samples can be expected in the presence of excess electrons originated from the nitrogen deficiency.

**Figure 4:**
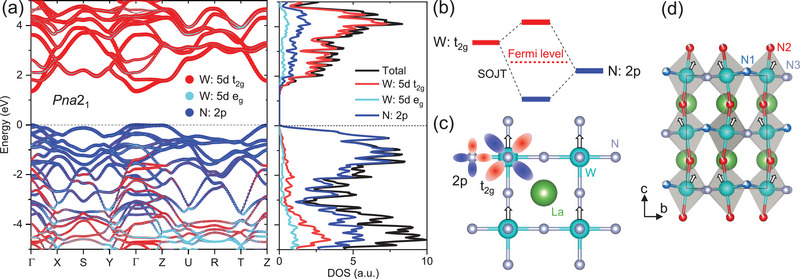
Band structure and mechanism for polar distortion of *Pna*2_1_‐LaWN_3_. a) Calculated band structure and DOS. b) Schematic of the W‐N hybridization for polar phase. c) Polar distortion in a nonpolar phase as denoted by arrows. The lobe‐shaped orbitals of N: 2*p_x_*‐2*p_y_* and W: 5*d_xy_* have two different phases of the associated wave function as denoted in red and blue, respectively. d) Atomic polarization in *Pna*2_1_‐LaWN_2.6_. The arrows denote the relative displacement of W to N in each octahedron.

We further explore electronic properties of *Pna*2_1_‐LaWN_3_. The band structure and density of states (DOS) are shown in **Figure** **4**a‐b. It is interesting to see that *Pna*2_1_‐LaWN_3_ has a direct bandgap of 1.36 eV without consideration of spin‐orbit coupling (SOC) (Figures S14–S15, Supporting Information), different from the metallic cubic$Pm\bar{3}m$‐ and *Pnma*‐LaWN_3_. The band edges around the Fermi level are mainly associated with the hybridized W: t_2g_‐N: p states (i.e., *pdπ* bonding/antibonding) (Figure 4b‐c), an analogy to that in most oxide perovskites.^[^ ^61^ ^]^ We find that the bandgap opening in polar‐LaWN_3_ is primarily due to the cooperative displacement of N relative to W that can lead to more efficient W‐N bonding/antibonding (Figure 4c and Figures S15–S16, Supporting Information). Such behaviors correspond to the second‐order Jahn‐Teller (SOJT) effect,^[^ ^62^ ^]^ which is responsible for bandgap opening, as seen in many perovskite materials (e.g., BaTiO_3_). In contrast, the nonpolar phases involve a large portion of W–N antibonding state at the Fermi level (Figure S15c). The effectively hybridized W–N bonds through the SOJT effect are believed to be essential to compete against the short‐range repulsion that favors the nonpolar symmetry, resulting in a polar phase with a large bandgap.

Given the definitively refined structure, the electric polarization of *Pna2*_1_‐LaWN_2.6_ can then be well explored, which involves complex octahedral distortions (Figure 4d). A prominent feature is the relative W‐N displacement along the N2‐W‐N2 line with a relative W shift of ≈0.035 Å from the N2‐N2 center (Figure S16, Supporting Information). A smaller displacement occurs along one of the N1‐W‐N3 lines with a shift of ≈0.016 Å towards N3, while the shift along the other N1‐W‐N3 line is negligible. Apparently, the electric dipoles of LaWN_2.6_ have a unique long‐range order along the *c‐*axis with a sinusoidal‐like chain of dipole orientation (Figure 4d). The thermal expansion coefficients of the material along the *c‐*axis are greater than those along other axes, suggesting the temperature sensitivity of its structure along the polarization direction, and no phase transition is involved in the 4 – 1100 K range, based on our variable‐T XRD experiments (Figures S19–S21 and Table S1, Supporting Information). Attempts to identify electric polarization of LaWN_2.6_ are unsuccessful by various measurements, due to the current leakage originated from the electron‐filling effect of nitrogen deficiency. Future work along this line is warranted to obtain stoichiometric sample and explore exciting ferroelectric properties for driving interesting functionality. Re‐treatment of LaWN_3‐*δ*_ in N_2_ gas at higher pressures (e.g., above 10 GPa) using diamond‐anvil cells and laser heating techniques may lead to stoichiometric LaWN_3_ with as‐predicted semiconducting properties. Alternatively, element substitution would provide another promising approach for obtaining semiconducting nitride perovskites; of particular interest is to replace W with Ta or Hf for reducing the number of 5d valence electrons, because 5d electrons are spatially so delocalized to be itinerant and unfavorable for opening a bandgap.

## 3 Conclusions

In summary, we have formulated an effective high‐P route for preparing oxygen‐free LaWN_3_ bulk samples with excellent crystallinity, leading to definitive identification of the crystal structure and composition of *Pna2*_1_‐LaWN_2.6_ with a polar symmetry for producing ordered dipoles along the *c‐*axis. Due to the electron‐filling effect, the nitrogen deficiency of this nitride is explored to have a profound influence on its phase stability and electronic properties. The strong W: t_2g_‐N: p hybridization in LaWN_3_ formed through the SOJT effect is revealed to be the driving force for polar distortion and bandgap opening. The well‐explored structure and properties of LaWN_3_ would offer important baseline data for understanding the complex relationships between the composition, chemistry, and crystal and electronic structures of ternary nitrides. Besides, the methodology established in this work can be extended to other nitride perovskites for discovering functional materials that may offer a fertile platform for exploring exotic phenomena at the frontier of condensed matter physics.

## 4 Experimental Section

### High P‐T Synthesis

High‐purity La_2_W_2_O_9_ and sodium amide (NaNH_2_) powders in a molar ratio of La_2_W_2_O_9_: NaNH_2_ = 1:10 was homogenously mixed for the synthesis of LaWN_3_. The excess NaNH_2_ was used to establish a nitrogen‐rich environment for a complete ammonization of La_2_W_2_O_9_. Note that La_2_W_2_O_9_ was obtained from a stoichiometric reaction between WO_3_ and La_2_O_3_ (i.e., in a molar ratio of 2:1) at 1400 °C for 10 h, using a muffle furnace. High P‐T synthesis were carried out using a DS 6 × 10 MN cubic press installed in the high‐P lab of SUSTech.^[^ ^63^ ^]^ Before the experiment, the powder mixture was compacted into a cylindrical pellet of 12 mm in diameter and 10 mm in height, which was then loaded in an *h*BN or Mo capsule and assembled with the preprepared cell parts. More experimental details can be found elsewhere.^[^ ^63^ ^]^ Due to the sensitivity of NaNH_2_ precursor to air, all the procedures were done in an argon‐filled glovebox to avoid possible contaminations. The synthesis was carried out in a wide temperature range of 600 to 2100 °C at 5 GPa for 10 – 30 min to prepare various samples with different crystallinities for comparative studies. The recovered nitride products were washed with distilled water to remove possible byproducts (e.g., NaOH) and unreacted NaNH_2_, followed by drying in an oven at 80 °C. For comparison purposes, we also performed the synthesis from a different reaction between oxygen‐free reactants of LaN, W, and NaNH_2_ in a molar ratio of 1:1:5 at 5 GPa and 1800 °C for 30 min.

### Characterization

The final products were checked by X‐ray diffraction (XRD) with a Cu K*α* radiation. Variable‐T XRD experiments were also carried out in the 4 – 1100 K temperature range to study possible phase transitions and lattice thermal expansion at ambient pressure. The neutron powder diffraction (NPD) measurement was performed at the neutron beamline of the China Mianyang Research Reactor (CMRR) and the wavelength of incident neutron beam is *λ* = 1.5925 Å. The XRD and NPD data were analyzed using the GSAS and FullProf programs, respectively. The scanning electron microscopy (SEM) equipped with energy‐dispersive X‐ray (EDX), Auger electron spectroscopy (AES), and transmission electron microscopy (TEM) experiments were conducted to study the morphologies, chemical composition, and crystal structure of as‐synthesized samples. AES measurements were conducted with a PHI 710 scanning Auger nanoprobe equipped with an argon ion source, and the oxygen detection limit of the instrument is ≈5%.^[^ ^64^, ^65^ ^]^ To investigate the variation of chemical composition of the sample with depth, the AES data was taken at different depths of 50, 100, 150, and 200 nm, respectively, etched by Ar^+^ ion beam with an etching rate of 23.8 nm min^−1^. X‐ray photoelectron spectroscopy (XPS) measurements were performed to study the binding energiey of the involved elements of sample.

The ultraviolet–visible (UV‐Vis) absorption and electrical transport experiments were performed to determine bandgap and resistivity of the sample, based on single‐crystal samples. The dc magnetization data was collected using a Quantum Design SQUID VSM magnetometer. The secondary harmonic generation (SHG) measurements were carried out to check the symmetry of the material with an incident laser wavelength of 880 nm. The thermogravimetric mass spectrometer (TG‐MS) measurements were carried out in air and argon, respectively, to investigate the sample's thermal stabilities. The Vickers hardness test was performed on a well‐sintered polycrystalline sample prepared at 5 GPa and 1200 °C for 30 min. The hardness of the sample was measured at different loads of 50, 100, 200, 500, and 1000 g. At each load, the measurement was repeated for more than five times to obtain statistic averages.

Attempts to identify the ferroelectric properties of our samples were unsuccessful by measuring the hysteresis loop of electric polarization against an external electric field using a ferroelectric analyzer, due to the current leakage originated from the electron‐filling effect of nitrogen deficiency that is unfavorable for obtaining robust ferroelectricity.

### Computational Methods

First‐principles calculations were performed within the density functional theory (DFT) by using the projector augmented wave (PAW) method with a plane‐wave basis set with an energy cutoff of 500 eV, as implemented in the VASP code.^[^ ^51^, ^52^, ^53^, ^54^ ^]^ The generalized gradient approximation (GGA) with the Perdew‐Burke–Ernzerhof (PBE) was used to treat the exchange‐correlation interactions for structural relaxations, while the HSE06 hybrid function was employed for the simulation of electronic structures.^[^ ^55^, ^56^ ^]^ The k‐point sampling was performed with a Monkhoust‐Pack scheme of a 11 × 9 × 11 grid with the Γ point included for orthorhombic cell. The convergence criteria for the Hellmann–Feynman force and total energy were set at 0.005 eV Å^−1^ and 10^−6^ eV, respectively, for structural relaxations. The phonon calculations were done with the help of the phonopy package.^[^ ^57^ ^]^ The crystal orbital Hamilton population (COHP) analyses were calculated with the LOBSTER package.^[^ ^66^ ^]^

The energies of the two competing structures of *R3c* and *Pna2*_1_ were calculated with PBE functional and based on a pristine 2 × 2 × 2 supercell (i.e., La_8_W_8_N_24_) with involvement of different numbers of nitrogen vacancies (i.e., 0, 1, 2, and 3), by which the supercell can be re‐scaled down to one formula unit of LaWN_3‐*δ*_, where *δ* corresponds to a normalized nitrogen vacancy number of 0, 1/8, 2/8, and 3/8. In addition, we also simulated the influence of nitrogen deficiency on the structural stability by adding free electrons into the dispersive conduction bands of LaWN_3_. In this case, a homogenous background charge was added for compensation of incorporated free electrons. During electron doping, the internal coordinates, shapes, and unit‐cell volume of crystal structure were freely relaxed; besides, the calculated results were weakly affected by volume relaxation. Note that there is no direct numerical equivalence between the number of nitrogen vacancy and free electron doped to the conduction bands. Our calculations indicate that nitrogen vacancy acts as a donor for supplying free electrons into conduction bands. The dipole moments of the electron‐doped systems were estimated from the calculations of Born effective charge using polar phases of *R3c* and *Pna2*_1_.

## Conflict of Interest

The authors declare no conflict of interest.

## Supporting information

Supporting Information

Supporting Information

## Data Availability

The data that support the findings of this study are available in the supplementary material of this article.
