# Supplementary material for: Polar Nitride Perovskite LaWN3‐δ with Orthorhombic Structure
Source: Adv Sci (Weinh). 2023 May 2;10(19):2205479. doi: 10.1002/advs.202205479 (PMC10323622; doi:10.1002/advs.202205479)
Supplement: Supplementary file 1 — Supporting Information [file ADVS-10-2205479-s001.pdf]

ADVANCED SCIENCE
Open Access

Supporting Information

for *Adv. Sci.*, DOI 10.1002/advs.202205479

Polar Nitride Perovskite $LaWN_{3-\delta}$ with Orthorhombic Structure

Xuefeng Zhou, Wenwen Xu, Zhigang Gui, Chao Gu, Jian Chen, Jianyu Xie, Xiaodong Yao, Junfeng Dai, Jinlong Zhu, Liusuo Wu, Er-jia Guo, Xiaohui Yu, Leiming Fang, Yusheng Zhao, Li Huang and Shanmin Wang**

Supporting Information

Polar nitride perovskite $LaWN_{3-\delta}$ with orthorhombic structure

Xuefeng Zhou[1,§], Wenwen Xu[1,§], Zhigang Gui[1,§], Chao Gu[1], Jian Chen[1], Jianyu Xie[1], Xiaodong Yao[1], Junfeng Dai[1], Jinlong Zhu[1], Liusuo Wu[1,2], Er-jia Guo[3], Xiaohui Yu[3], Leiming Fang[4], Yusheng Zhao[1], Li Huang[1,2*], Shanmin Wang[1,2*]

[1]*Department of Physics & Academy for Advanced Interdisciplinary Studies, Southern University of Science & Technology, Shenzhen, Guangdong, 518055, China*
[2]*Quantum Science Center of Guangdong-Hongkong-Macao Greater Bay Area, Shenzhen, Guangdong, 518055, China*
[3]*Beijing National Laboratory for Condensed Matter Physics and Institute of Physics, Chinese Academy of Sciences, Beijing 100190, China*
[4]*Key Laboratory for Neutron Physics, Institute of Nuclear Physics and Chemistry, China Academy of Engineering Physics, Mianyang 621999, China*

*E-mail: huangl@sustech.edu.cn (L. Huang); wangsm@sustech.edu.cn (S. Wang)

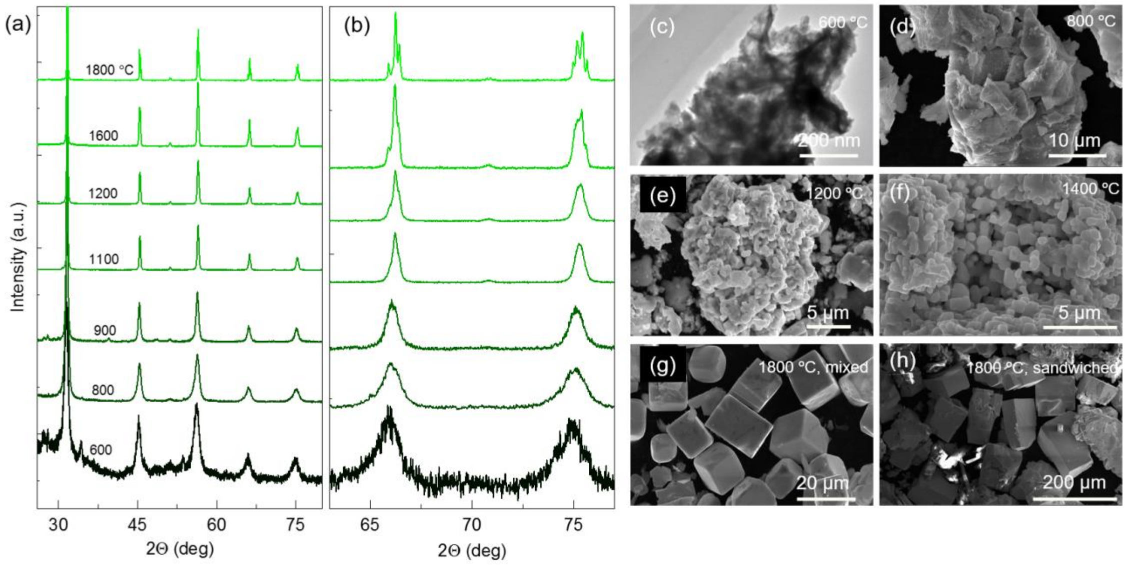

Fig. S1. XRD patterns and SEM images of as-prepared $LaWN_{3-\delta}$. (**a**) XRD patterns of samples synthesized at 5 GPa and different temperatures from homogenously mixed reactants of $La_2W_2O_9$ and $NaNH_2$. (**b**) Enlarged portion of XRD patterns in (a). (**c**) – (**d**) Selected TEM and SEM images of samples synthesized at 5 GPa and 600, 800, 1200, 1400, and 1800 ºC, respectively. The image in (c) is a TEM image and those in (d)-(f) are SEM images. (**h**) Large single-crystal $LaWN_{3-\delta}$ synthesized at 5 GPa and 1800 ºC from a sandwiched assembly of reactants (i.e., one $La_2W_2O_9$ layer is sandwiched by two layers of $NaNH_2$).

To overcome the disadvantages of previous high P-T synthetic approaches for nitride preparation with involvement of highly explosive azides (e.g., NaN_3) [1-3], we formulate a favorable route for synthesizing $LaWN_{3-\delta}$ from the reaction between $La_2W_2O_9$ and $NaNH_2$ at 5 GPa and temperatures of 600 – 2100 ºC, given by

$$La_2W_2O_9 + 9NaNH_2 = 2LaWN_3 + 9NaOH + 3NH_3 \qquad (1),$$

where the byproduct NaOH was identified by XRD measurement, and a strong odor released from the recovered sample suggests the presence of NH_3 gas. To eliminate impurities, excess $NaNH_2$ was included in the reaction with a molar ratio of $La_2W_2O_9$: $NaNH_2$ = 1: 10. The recovered sample can readily be purified by washing with water to remove both the soluble byproduct NaOH and unreacted $NaNH_2$ for obtaining phase-pure samples (Figs. S1-S3).

Judging from the XRD peak broadening, the sample synthesized below 1200 ºC should involve severe lattice distortion or low crystallinity in nanocrystalline forms. Indeed, the sample synthesized at 600 ºC is really nanocrystalline without well-defined crystal shape. The lattice distortion can be identified from TEM measurements in Fig. S2.

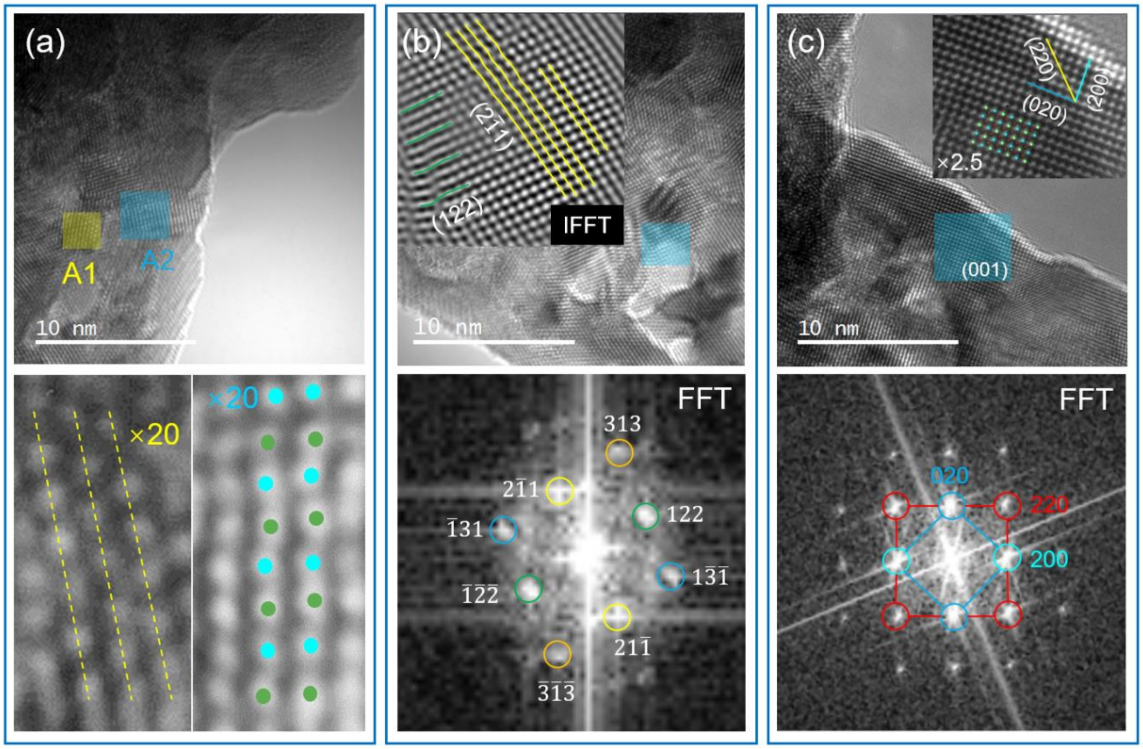

Fig. S2. TEM observations of $LaWN_{3-\delta}$ synthesized at 5 GPa and a relatively low temperature of 600 ºC for 30 min. (**a**) A typical TEM image. The bottom panel show two enlarged portions of A1 and A2 to demonstrate the lattice distortions of W and La. (**b**) IFFT and FFT patterns of a selected area of TEM image as hatched in blue. (**c**) FFT patterns of a selected area of TEM taken with an incident electron beam along the [001] direction. Inset in the top-right corner of the upper panel shows an enlargement of selected area (as hatched in blue) with the lattice fringes associated with the (010), (100), and (110) crystallographic planes.

Clearly, the disordered lattice can be identified as shown in Figs. S2(a)-S2(b), which should be responsible for the largely diffused electron diffraction spots [bottom panel of Fig. S2(b)]. Note that the 110-diffraction spot is absent in this sample [bottom panel of Fig. S2(c)], indicating the disordered N atoms.

According to our structural refinement (see Table 1 of main text), the polar displacement of $LaWN_{3-\delta}$ is closely associated with nitrogen atoms that are insensitive to electron diffraction, which is different from the cases of conventional oxide ferroelectrics ABO_3 (e.g., $BiFeO_3$ and $BaTiO_3$) with ferroelectric polarization of metal B atoms (e.g., Fe and Ti). Therefore, it is challenging to directly observe atomic polarization in $LaWN_{3-\delta}$ by TEM.

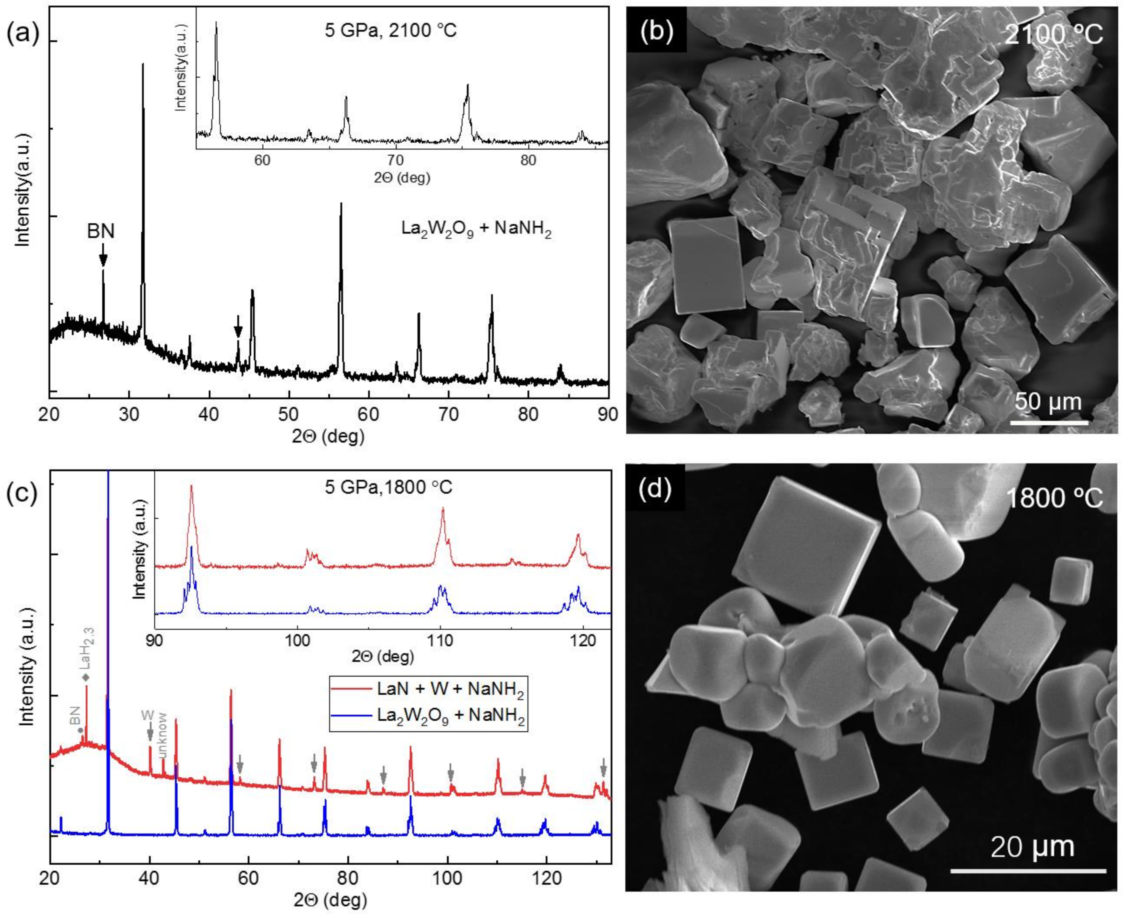

Fig. S3. Growth of large single-crystal samples at 2100 ºC and an oxygen-free reaction route for synthesizing $LaWN_{3-\delta}$. (a) – **(b)** XRD pattern and SEM image of $LaWN_3$ sample synthesized from homogenously mixed reactants of $La_2W_2O_9$ and $NaNH_2$ at 5 GPa and 2100 ºC. (**c**) – (**d**) XRD pattern and SEM image of $LaWN_3$ sample synthesized from a different reaction between the oxygen-free reactants of LaN, W, and $NaNH_2$ at 5 GPa and 1800 ºC. Also plotted in (c) is an XRD pattern of the sample prepared from the reaction between $La_2W_2O_9$ and $NaNH_2$ under the same high *P*-*T* conditions. Insets in (a) and (c) are the enlarged portions of the corresponding XRD patterns to show the details of peaks in the high-2Θ range.

For the sample synthesized from the reaction between LaN, W, and $NaNH_2$, our AES measurements show that the La:W molar ratio of the selected spot in the interior of the sample is occasionally observed to deviate from 1:1, indicating a compositional inhomogeneity. Similar phenomenon has also been previously observed in thin-film $LaWN_3$ (see details in ref. [4]). In contrast, for the sample synthesized from the reaction between $La_2W_2O_9$ and $NaNH_2$, the La and W are more uniformly distributed with a nearly same ratio of La:W = 1:1 [see Fig. S4(h)].

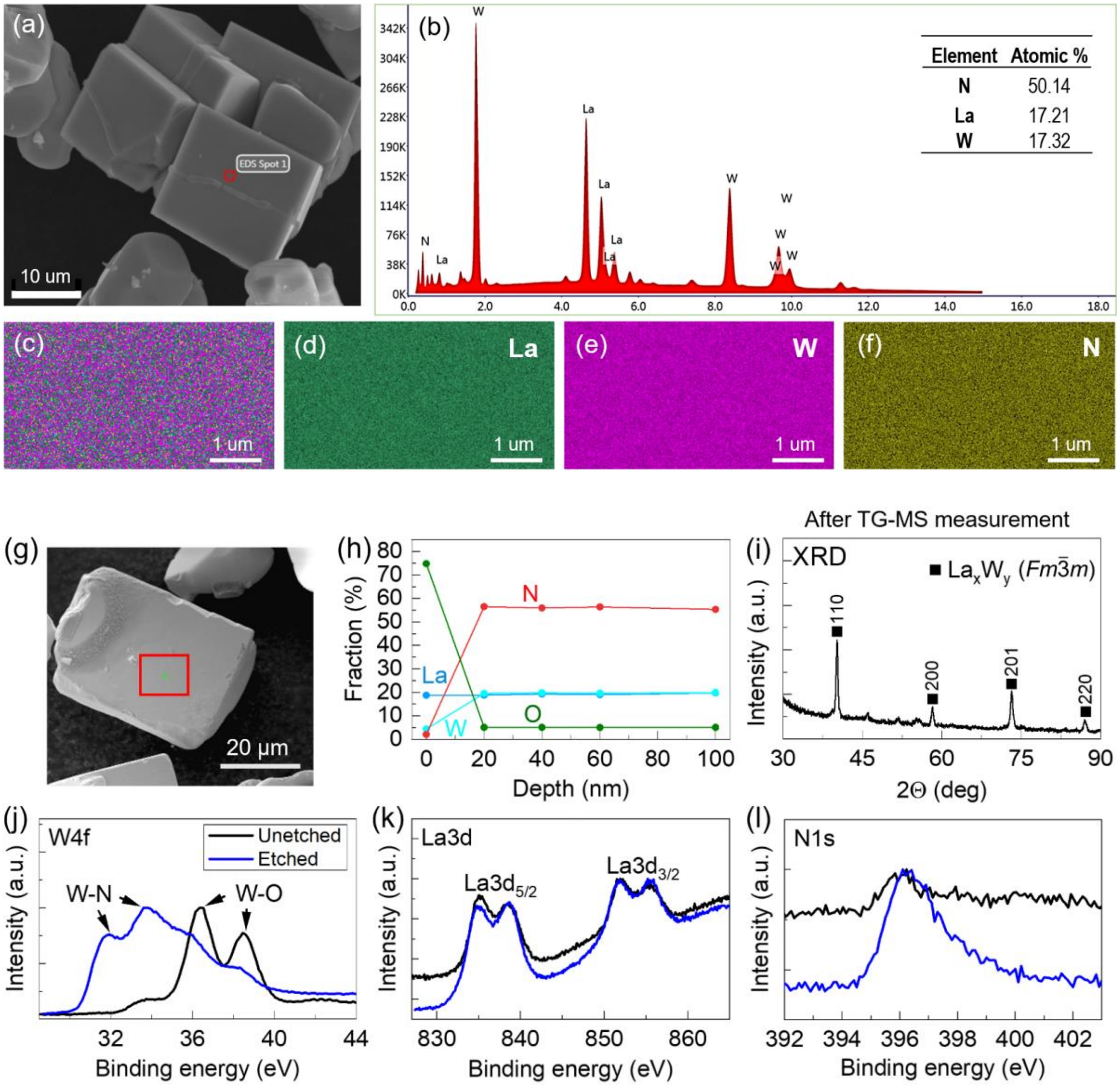

Fig. S4. Composition determination by EDX, element mapping, AES, XRD, XPS, and thermally-induced decomposition experiments based on single-crystal $LaWN_{3-\delta}$ synthesized at 5 GPa and 1800 ºC for 30 min. (**a**) SEM image. (**b**) EDX spectrum of the selected area of the sample in (a), as denoted by the white lines. (**c**) - (**f**) Element mapping of all the constituent elements, La, W, and N, respectively. (**g**) - (**h**) AES measurements based on the framed area of single-crystal sample [see red rectangle lines in (g)]. (h) Determined compositional ratios of the involved elements as a function of etching depth. (**i**) XRD pattern of the recovered product after TG-MS experiment by treating $LaWN_{3-\delta}$ in argon. (**j**)-(**l**) W4f, La3d and N1s XPS spectra of $LaWN_{3-\delta}$ before and after surface etching treatment with Ar^+ ion beam, as denoted by black and blue lines, respectively.

Our EDX measurement shows the sample is compositionally constituted by La, W, N in a molar ratio in the range of ~1:1:2.8 – 1:1:3.5. Because of the different sensitivities of metals and N to electrons, the EDX method cannot achieve an accurate determination of the ratio of these elements.

In Fig. S4(h), the sample surface has a high oxygen concentration of ~74%, in striking contrast to that of nitrogen with a low concentration of ~2%. With the increase of etching depth above 20 nm, the oxygen fraction is quickly decreased to an asymptotic value of ~5%, which is nearly the detection limit of the instrument for oxygen[5-6], similar to that of reported for oxygen-free thin-film $LaWN_3$[4]. Note that the elemental detection limit of AES instrument can be calibrated using standard samples such as TiN and gold. Meanwhile, nitrogen has a sharp increase from ~2% to ~56%, and both the La and W are leveled off at the value of ~19%. Nevertheless, AES measurements clearly indicate that there exists a 20-nm-thick oxide layer on the surface of sample grains, which explains the presence of oxygen impurity in both the EDS and XPS spectra (see Fig. 2 of main text). Such surface oxide layer probably forms during purification by washing with water or sample drying in oven at 80 ºC (see Experimental details in the main text). In fact, the thin oxidation layers occur inevitably for most transition-metal (TM) nitrides, especially the W-N compounds, referring to our recent work on cubic tungsten nitrides[7]. Because the surface layer is thin enough, which does not alter the bulk properties of nitrides.

In addition, the decomposition of the titled material in Ar gives rise to final products of metal W or a W-La alloy (i.e., La_xW_y) with a rocksalt structure having a refined lattice parameter of a = 4.4818 Å[Fig. S4(i)]. To further confirm the presence of a surface oxide layer, we performed XPS measurements on the pretreated sample by surface etching with Ar^+ ion beam [Figs. S4(j)-S4(l)]. Clearly, the etched sample has a profoundly reduced intensity of XPS W4f doublet lines associated with W-O, indicating a negligible concentration of oxygen in the interior of crystals, although very small doublets still remain, similar to that of cubic tungsten nitrides as reported in our previous work[7]. The XPS La3d lines of both unetched and etched samples are nearly same, implying a similar valence state of La in both $LaWN_{3-\delta}$ and its surface La-O compound [Fig. S4(k)]. The increased intensity of N1s line in the etched sample may be due to the decreased oxygen concentration [Fig. S4(l)].

Overall, all these measurements strongly suggest that our final product is really oxygen-free $LaWN_{3-\delta}$, rather than an oxynitride of $LaWO_xN_y$.

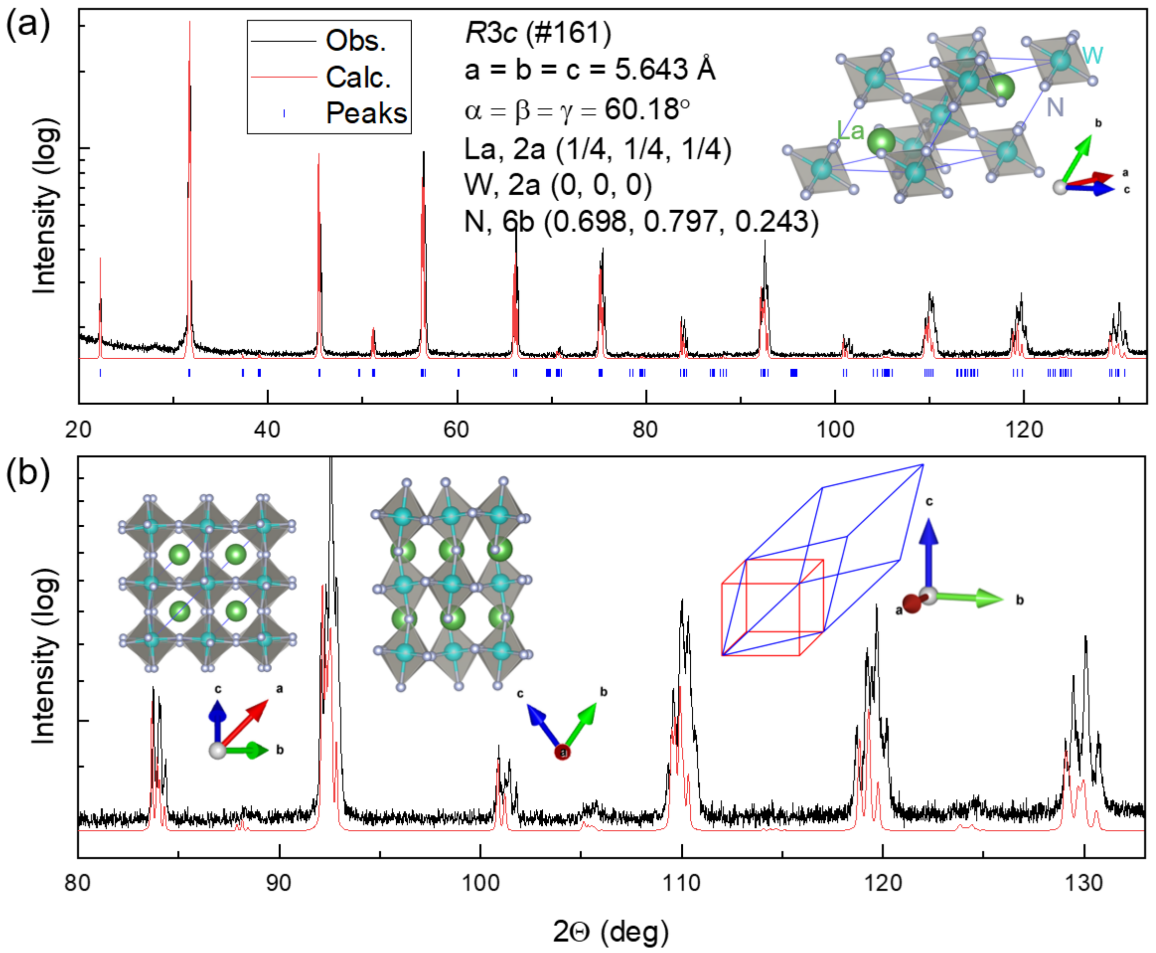

Fig. S5. Refined XRD using the rhombohedral *R3c* structural model. (**a**) Comparison between the experimental XRD data and simulated pattern using the refined *R*3*c* structure. The refined crystal structure and lattice parameters are given in (a). (**b**) Enlarged portion of a high-2Θ region of the XRD pattern to show details. A polyhedral view of the crystal structure is given as an inset (left). The relationship between the pristine cubic cell and *R3c* cell is also plotted (right), as denoted in red and blue, respectively. The ideal lattice parameters of *R3c* are $a' = \sqrt{2}a$ and $\alpha = \beta = \gamma = 60°$, where a is the lattice parameter of the pristine cubic cell. The XRD pattern simulations are performed using a Cu target with a doublet of the $K\alpha_1$and $K\alpha_2$ radiations having incident wavelengths of $\lambda_{K\alpha_1}$= 1.54056 Å and $\lambda_{K\alpha_2}$= 1.54440 Å, respectively.

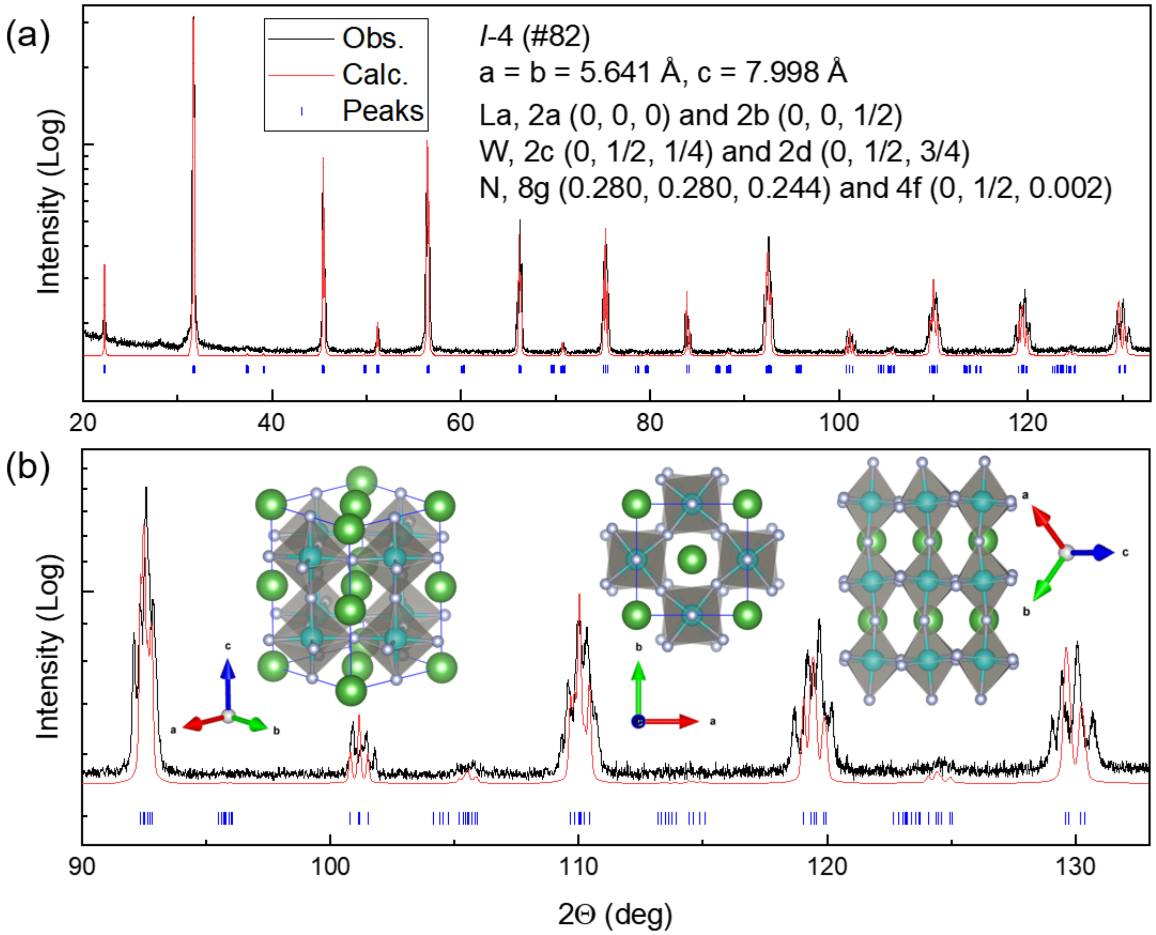

Fig. S6. Refined XRD using the tetragonal $I\bar{4}$ structural model. (**a**) Comparison between the experimental XRD data and simulated pattern using the refined $I\bar{4}$ structure. The listed lattice parameters for $I\bar{4}$ structure in (a) are associated with the best refinements of both XRD and NPD patterns. (**b**) Enlarged portion of a high-2Θ pattern to show details. Polyhedral views of the crystal structure of $I\bar{4}$ are given as insets. The ideal lattice parameters of $I\bar{4}$ are $a' = \sqrt{2}a$ and $c' = 2a$, where a is the lattice parameter of the pristine cubic cell. The XRD pattern simulations are performed using a Cu target radiation.

Although the low-angle XRD peaks (i.e., 0 – 60º) can be matched with the calculated using the structural model of $R3c$ or $I\bar{4}$, apparent discrepancies occur in the high-2Θ range (i.e., above 90º) (Figs. S5-S6). Our XRD refinements show that all the diffraction peaks can be excellently matched with the calculated using the model of $Pna2_1$, $Pmc2_1$, or $Pnma$. If $R3c$ or $I\bar{4}$ phase is involved, large mismatches will appear for the high-2Θ peaks, which would profoundly deteriorate the figure-of-merit of refinement. In addition, TEM and SEM observations indicate that the sample is microstructurally uniform without phase segregation. These evidences can completely exclude the possibility of coexistence of $R3c$ and $I\bar{4}$ phase.

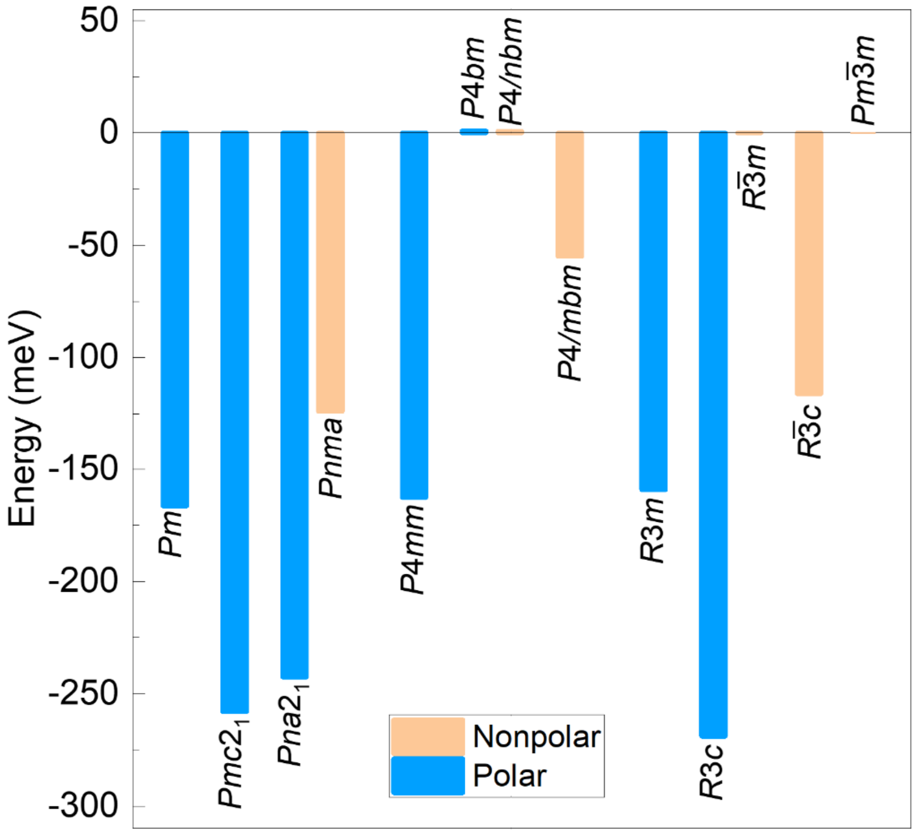

Fig. S7. Calculations of the total energy of various structures relative to *Pm*$\bar{3}$*m*-$LaWN_3$ with PBE functional per formula unit. The detailed crystal structures of those structural models are also given separately and available online in CIF format.

In a recent report by Talley *et al.*, thin-film $LaWN_{3-\delta}$ was prepared at 900 ºC by a physical vapor deposition (PVD) method and shows anomalous peak broadening, similar to the situations of our samples prepared below 1200 ºC at 5 GPa (see Fig. S1), due to the low crystallinity of samples. In this case, the broadened peaks are actually composed of a number of subpeaks, and it is inappropriately to deem such broadened peaks as single reflection lines for symmetry analysis, which would certainly lead to structural misassignment. In striking contrast, the well-crystallized $LaWN_{3-\delta}$ samples are obtained above 1400 ºC at 5 GPa in our high-P synthesis (Fig. S1), showing complicated peak splitting of such broadened peaks of low-T samples, by which *R*3*c* can be readily excluded (Fig. S5), although it is energetically more favorable as previously predicted[8]. Our further calculations show that *P*4*mm*, *Pmc*2_1, and *Pna*2_1 with polar symmetries are the most probable structural candidates for $LaWN_3$ (Fig. S7).

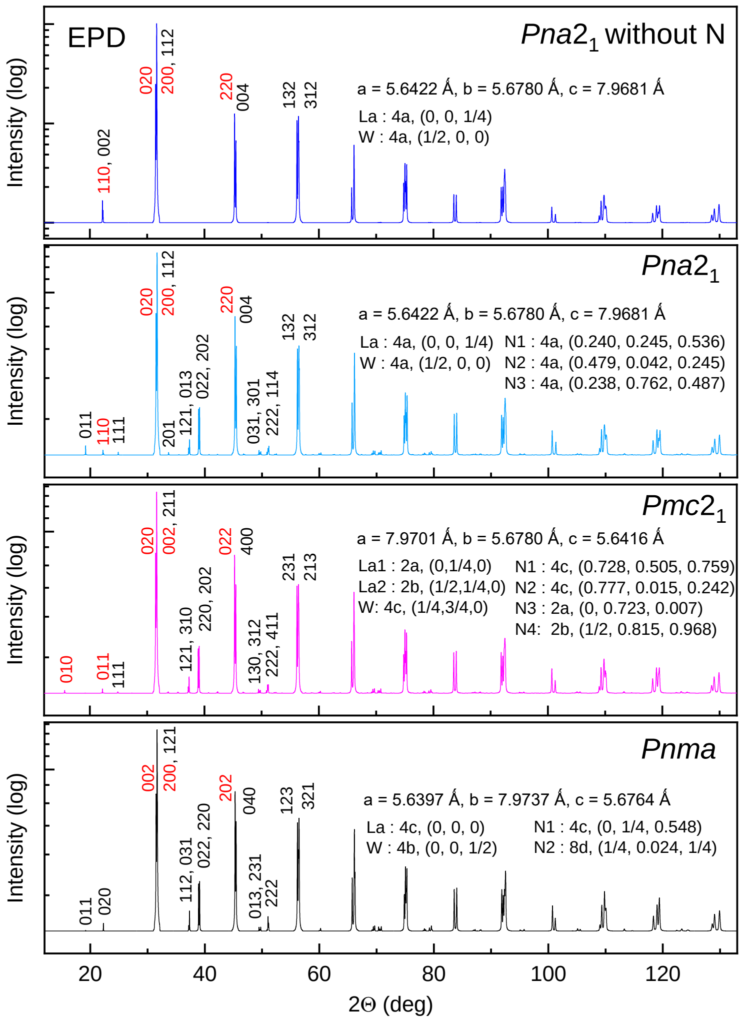

Fig. S8. Simulated electron powder diffraction (EPD) using the refined structural models of *Pnma*, *Pmc*2$_1$, *Pna*2$_1$, and *Pna*2$_1$ without N. For the TEM image with lattice fringes lying in the (010) plane for *Pnma*, the (100) plane for *Pmc*2$_1$, or the (001) plane for *Pna*2$_1$ (see Fig. 1 in the main text), the fast Fourier transformation (FFT) can only produce a subset of selected EPD peaks of {002, 200, 202…}, {010, 011, 020, 002, 022…}, or {110, 020, 200, 220…}, respectively. Insets in each panel are the refined lattice parameters based on the analysis of both the NPD and XRD data and they are used for the simulations of EPD patterns. The top panel is the simulated EPD pattern of *Pna*2$_1$ by removal of all the N atoms, and the completely same EPD pattern can also be obtained using either the *Pnma* or *Pmc*2$_1$ model with the absence of N atoms. This indicates that the smaller peaks around 2Θ = 20° arise from the N atoms, by which the structural ambiguities between them can be discerned.

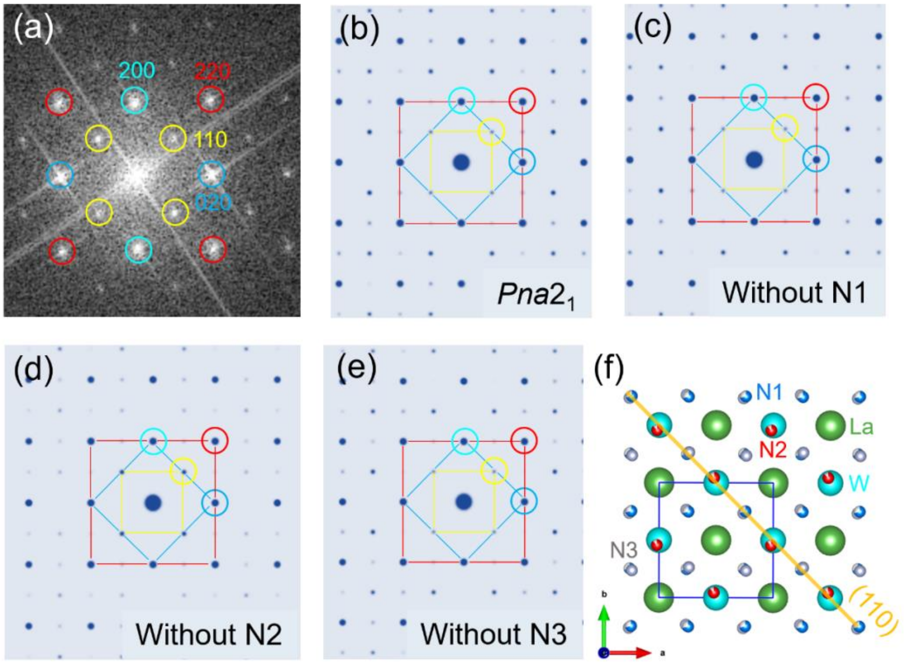

Fig. S9. Contributions of N1, N2, and N3 to the electron diffraction based on the refined *Pna2*$_1$ structure. (**a**) Observed single-crystal electron diffraction (SED) pattern along the [001] direction by an FFT of a selected region of the TEM image in Fig. 1(c) of the main text. (**b**) Simulated SED pattern using the refined *Pna2*$_1$. (**c**) – (**e**) Simulated SED patterns based on the refined *Pna*2_1 by removal of N1, N2, and N3, respectively. (**f**) Crystal structure of the refined *Pna*2_1. The N2 atoms (red) lie in the (110) plane, hence profoundly affecting the intensity of the 110-diffraction spot.

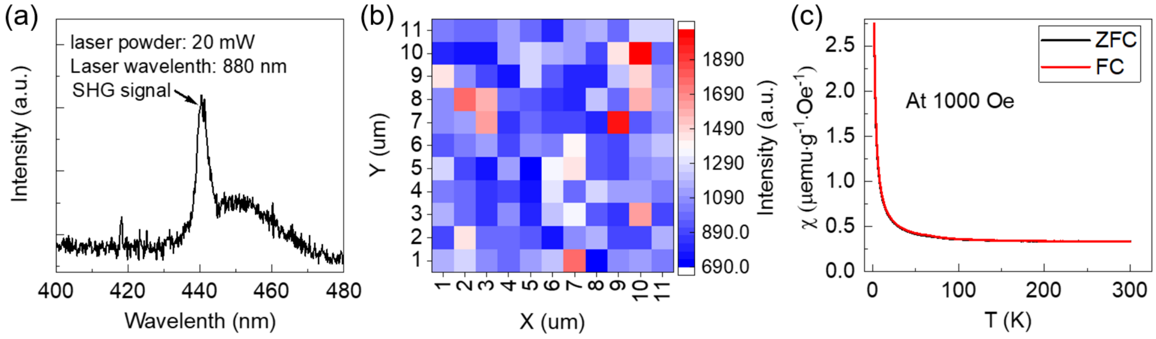

Fig. S10. Noncentrosymmetry and paramagnetism of $LaWN_{3-\delta}$. (**a**) Secondary harmonic generation (SHG) measurement of a single-crystal sample. The incident laser radiation has a wavelength of 880 nm. (**b**) SHG mapping for a well-sintered polycrystalline sample within a selected area of 100 um^2. (**c**) Magnetization measurement at 1000 Oe. Clearly, the material shows a typical of paramagnetic behavior.

To determine the noncentrosymmetric nature of our sample, we performed SHG measurements on both single-crystal and sintered polycrystalline samples with a laser wavelength and power of 880 nm and 20 mW, respectively, with an integration time of 30 - 60 s. It is noted that the SHG intensity strongly depends on crystallographic orientation of the involved crystals. For a polycrystalline sample, measurements along the polarization direction (e.g., the *c*-axis of $LaWN_{3-\delta}$) often give rise the strongest SHG intensity, while deviations from this direction will lead to a large decease of SHG intensity, as seen in Fig. 10(b). The spatial variation of SHG intensity can exclude the surface effect as the broken symmetry of surfaces may also produce a nonzero SHG signal.

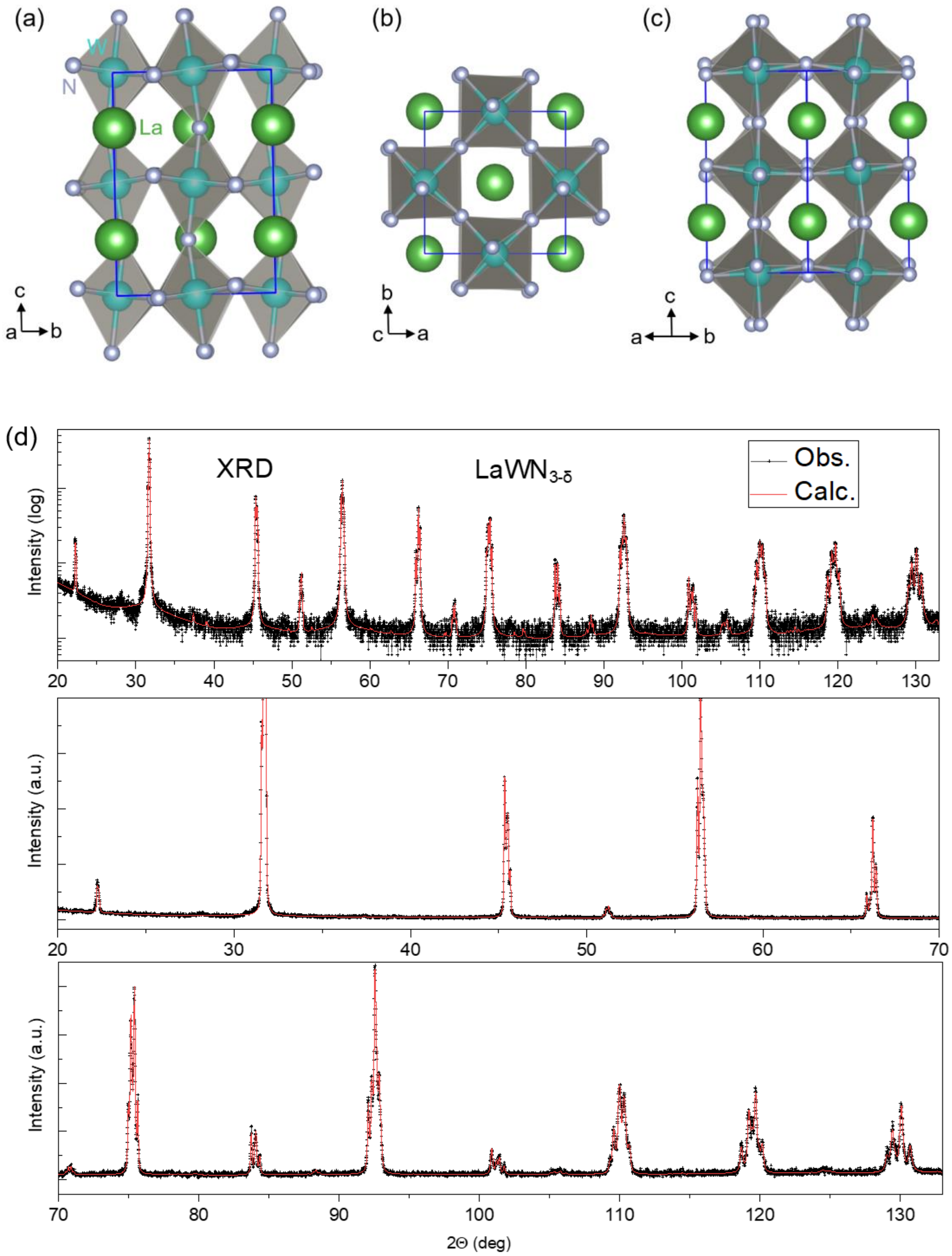

Fig. S11. Polyhedral views of the final *Pna*2$_1$ structure of $LaWN_{3-\delta}$ and the refined XRD pattern. (**a**)-(**c**) Different views of crystal structure. (**d**) Refined XRD pattern (full pattern, top panel) with enlarged portions in the 20-70º and 70-133º ranges (two bottom panels).

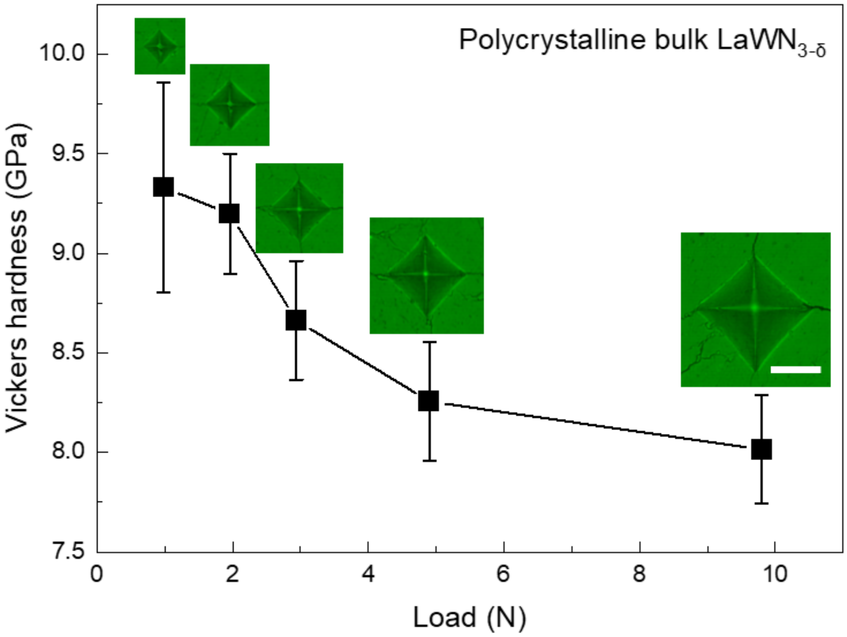

Fig. S12. Vickers hardness measurements based on a high-density polycrystalline $LaWN_{3-\delta}$ sample. The bulk sample was prepared from a sintering of purified sample powders at 5 GPa and 1200 ° for 30 min. Insets are typical images of indentations at different loads and the images have a same scale bar of 20 μm.

Compared with the rocksalt transition-metal nitrides[9], the current material is much more brittle with a very low toughness, indicating nearly complete ceramic behaviors. The nanoindentation measurements were also performed, showing a similar hardness and toughness.

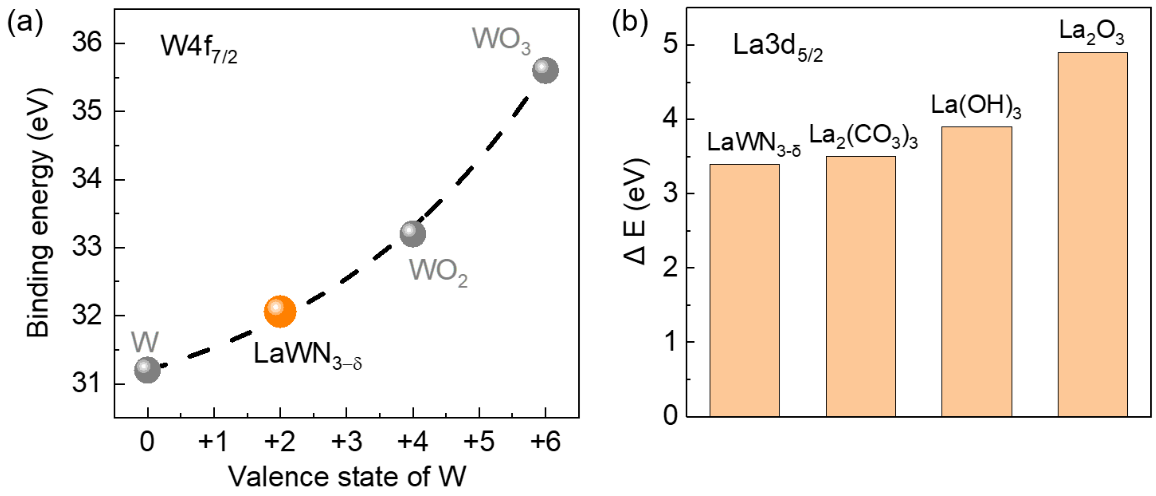

Fig. S13. Valence states of W and La in our $LaWN_{3-\delta}$ sample by analysis of the XPS data. (**a**) Determination of the oxidation state of W in our high-P sample, using the relationship between the $W4f_{7/2}$ binding energy and valence state, as established based on the reported data of the known compounds of W, WO_2, and WO_3[10]. (**b**) Energy split (ΔE) of the $La3d_{5/2}$ doublet of the sample. The cases of a few different La-bearing compounds (e.g., $La_2(CO_3)_3$, $La(OH)_3$, and La_2O_3) [11-12] are also plotted for comparison.

Each of the La: $3d_{5/2}$ and $3d_{3/2}$ states split into a doublet, giving a similar energy split of $\Delta E \approx 3.4$ eV [see Fig. 2(a) in the main text], close to that of purely ionic La^{3+} (e.g., $La_2(CO_3)_3$) but more than 25% smaller than that of La_2O_3 with certain covalency.

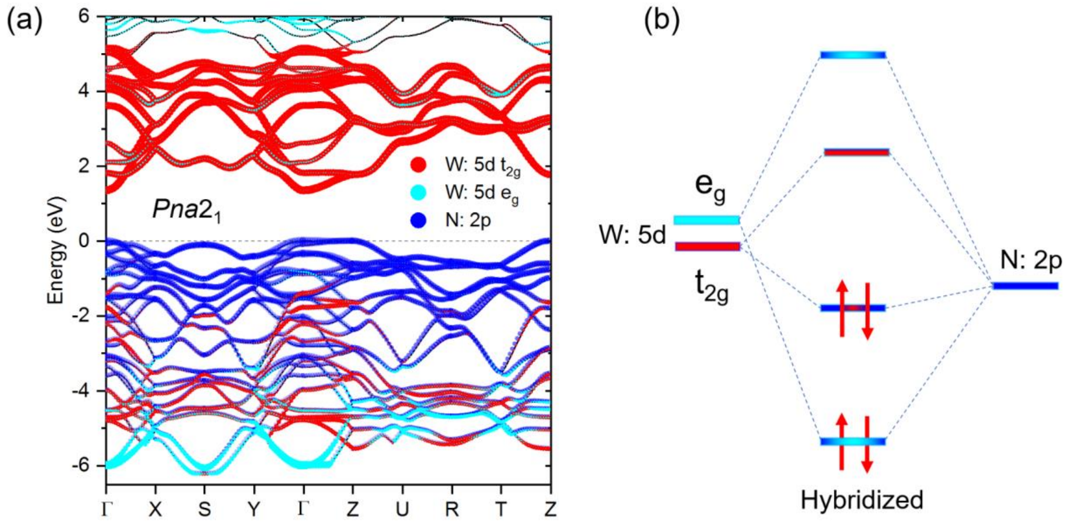

Fig. S14. Calculated electronic band structure and schematic of hybridization of W:5d and N: 2p for band splitting in *Pna*2$_1$-LaWN$_3$. (**a**) Simulated band structure of *Pna*2$_1$-LaWN$_3$. (**b**) Schematic illustration of orbital splitting due to the hybridization of W: 5d and N: 2p states. The hybridized orbitals in (a) split into four bands, corresponding to the simulated bands in (a). Such four bands are separated into two groups by a direct bandgap of 1.36 eV. The two upper bands are empty conduction bands (i.e., antibonding states), whereas the two bottom bands are fully filled valence bands (i.e., bonding states). It is noted that our additional calculations on the realistic LaWN$_{2.6}$ sample show a nearly zero bandgap, based on the PBE method and experimentally refined crystal structure, because of the defect-induced effect.

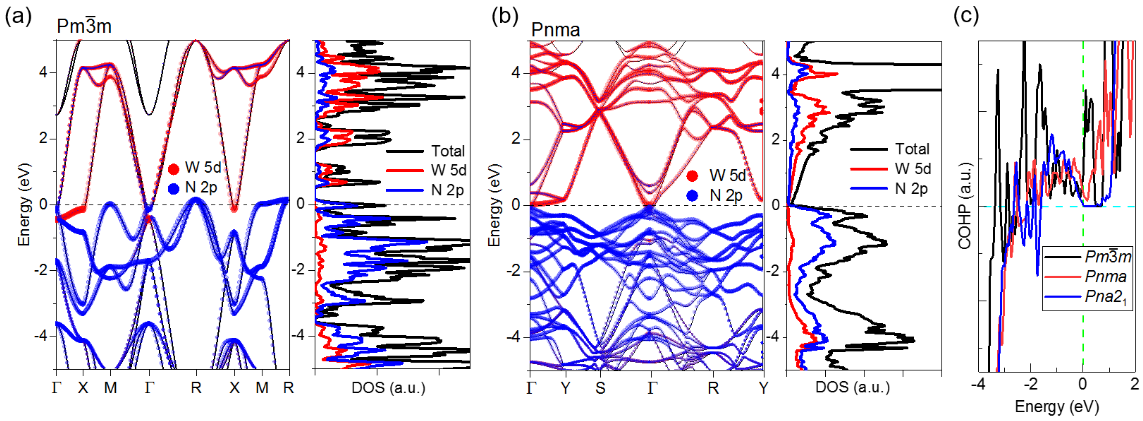

Fig. S15. Band structure, DOS, and crystal orbital Hamilton population (COHP) calculations for $LaWN_3$. (**a**) Band structure and DOS of $Pm\bar{3}m$-$LaWN_3$. (**b**) Band structure and DOS of *Pnma*-$LaWN_3$. (**c**) COHP calculations of nonpolar $Pm\bar{3}m$ and *Pnma* and polar $Pna2_1$ structures. There are considerable antibonding states near the Fermi level for the nonpolar phases, indicating they are structurally unstable.

Analysis shows that the states in nonpolar structures involve a large portion of W-N antibonding states at the Fermi level, which is strikingly different from the case in polar structures. The structural instabilities of nonpolar-$Pm\bar{3}m$ and -*Pnma* phases are closely related to the filling of W-N antibonding states, which also leads to the closure of bandgap. Polar distortions from W and N can efficiently alleviate such antibonding states at the Fermi level, which lowers the total energy and opens a bandgap by pushing down the top of valence bands and pushing up the bottom of conduction bands. Such picture is consistent with the SOJT effect [Fig. 4(b)].

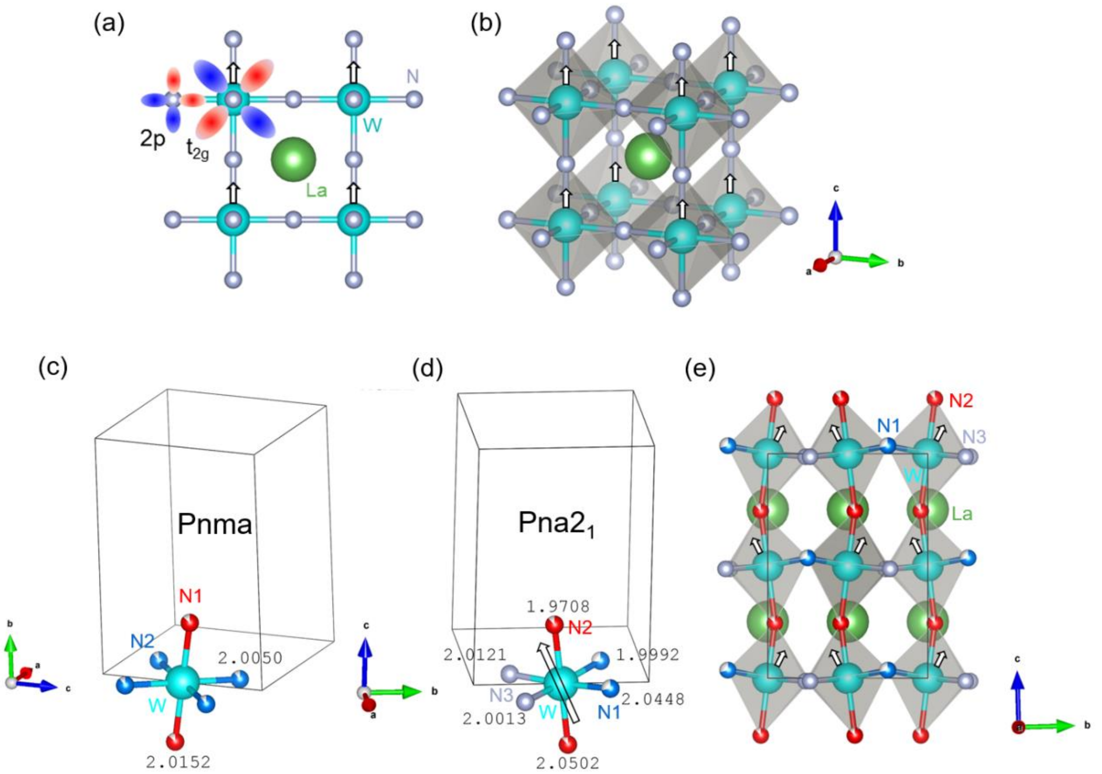

Fig. S16. Origins of atomic polarization in *Pna*2$_1$-LaWN$_3$. (**a**) Schematic diagram of the W: 5d t$_{2g}$ – N: p bonding states in the nonpolar symmetries (e.g., $Pm\bar{3}m$ or *Pnma*). The lobe-shaped orbitals of N: 2p_x-2p_y and W: 5d_{xy} have two different phases of the associated wave function as denoted in red and blue, respectively. (**b**) Polar distortion along the cell axis of a nonpolar structure with a shift of the center W atoms relative to the N coordination atoms. (**c**) – (**d**) Refined W-N distances in each WN$_6$ octahedron, based on the *Pnma* and *Pna*2$_1$ structures, respectively. In nonpolar-*Pnma*, all the W-N are the same with a value of $d_{\text{W-N}}$ = 2.0050 Å. In polar-*Pna*2$_1$, all the $d_{\text{W-N}}$ is different, and the most remarkable shift occurs along the W-N2 bond direction. The big arrow denotes the overall electric polarization of the involved WN$_6$ octahedra. (**e**) Atomic polarization in our *Pna*2$_1$-LaWN$_{2.6}$ sample, as determined by our structural refinement. The arrows in (a), (b), (d) and (e) denote the directions of polar distortions.

It is noted that the optimized structure by DFT calculations (i.e., denoted by S-DFT) does not involve vacancies, which gives rise to a sizeable bandgap [Fig. S14(a)] and a large electric polarization [Fig. S17(a)]. However, for the experimentally refined structure (i.e., denoted by S-EXP), our calculations show that its bandgap completely vanishes. The large difference in their calculated electronic properties can be attributed to their crystal structures with distinct polar displacements and nitrogen octahedral rotations [Figs. S14-S15 and Fig. 3 of the main text]. In particular, the dipole moment from S-EXP is less than 1/7 of that of S-DFT, which should be mainly responsible for the bandgap closure of S-

EXP. Such bandgap-polarity correlation is also consistent with the SOJT picture (see detailed in the main text). Another extreme example is *Pnma* structure with a zero bandgap [Fig. S15(b)], which can be approximately viewed as a nonpolar $Pna2_1$ structure. Another difference between S-EXP and S-DFT is that the in-phase rotation of nitrogen octahedra; however, the rotation in S-EXP is greatly decreased when compared to that of S-DFT. We note here that no N vacancy is involved in our calculations using a 20-atom supercell. But the S-EXP includes the averaged structural relaxations and the contribution of N vacancies is also involved. In Fig. S17, we also show that there is a similar scenario if free electrons are doped into $LaWN_3$-$Pna2_1$. This indicates that the existence of N vacancies can greatly affect both the electronic and structural properties of host $LaWN_3$, which accounts for its bandgap reduction and in-phase octahedral rotation.

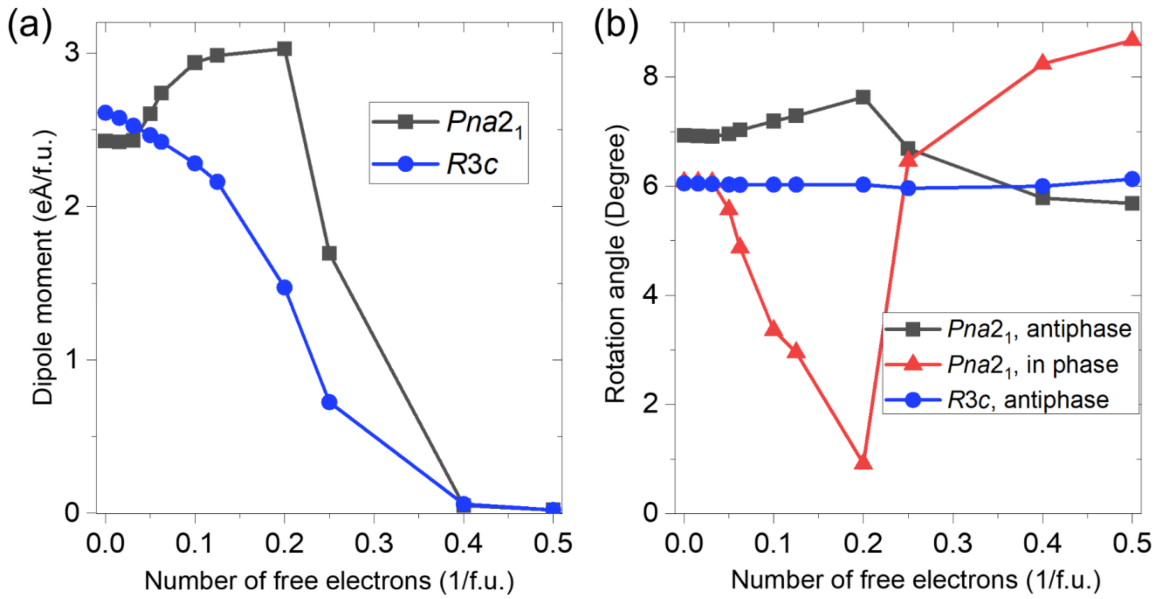

Fig. S17. Structural distortion evolution vs. the number of free electrons that are added to the conduction bands of *R*3*c* and *Pna*2$_1$ phases. (**a**) Dipole moment vs. free electron doping per formula unit for *R*3*c* and *Pna*2$_1$ structures. The dipole moment was determined through calculations of the Born effective charge using *R*3*c* and *Pna*2$_1$, respectively. (**b**) Oxygen octahedral rotation angle vs. free electron doping in *R*3*c* and *Pna*2$_1$ structures. Antiphase rotation angle (blue dots) for *R*3*c*; antiphase rotation angle (black square) for *Pna*2$_1$; in-phase rotation angle (red triangles) for *Pna*2$_1$. The rotation angles are estimated by comparing the crystal structures of *Pna*2$_1$ and R3c with that of $Pm\bar{3}m$ phase without involving rotation, respectively.

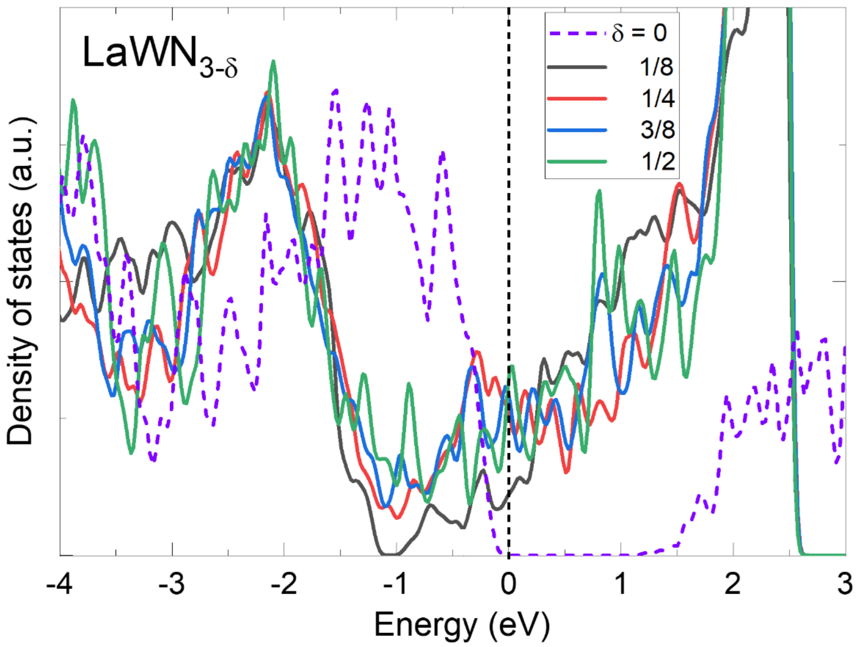

Fig. S18. Total density of states (DOS) of a 2 × 2 × 2 supercell (i.e., containing 8 formula units) with zero, one, two, three, and four nitrogen vacancies, corresponding to nitrogen vacancy concentrations of δ = 0, 1/8, 1/4, 3/8, and 1/2 in $LaWN_{3-\delta}$. The vertical dotted line indicates the positions of their Fermi levels. For the pristine case of $LaWN_3$, the fermi level is set to the valence band maximum.

From the DOS calculations, the pristine $LaWN_3$ displays a considerable bandgap of $E_g \approx 1.5$ eV, showing an insulating state. However, as the concentration of N vacancy increases, there are clearly large accumulations of DOS at the Fermi level, which tune the system from its pristine insulating to metallic state, accounting for the enhanced metallicity in the system.

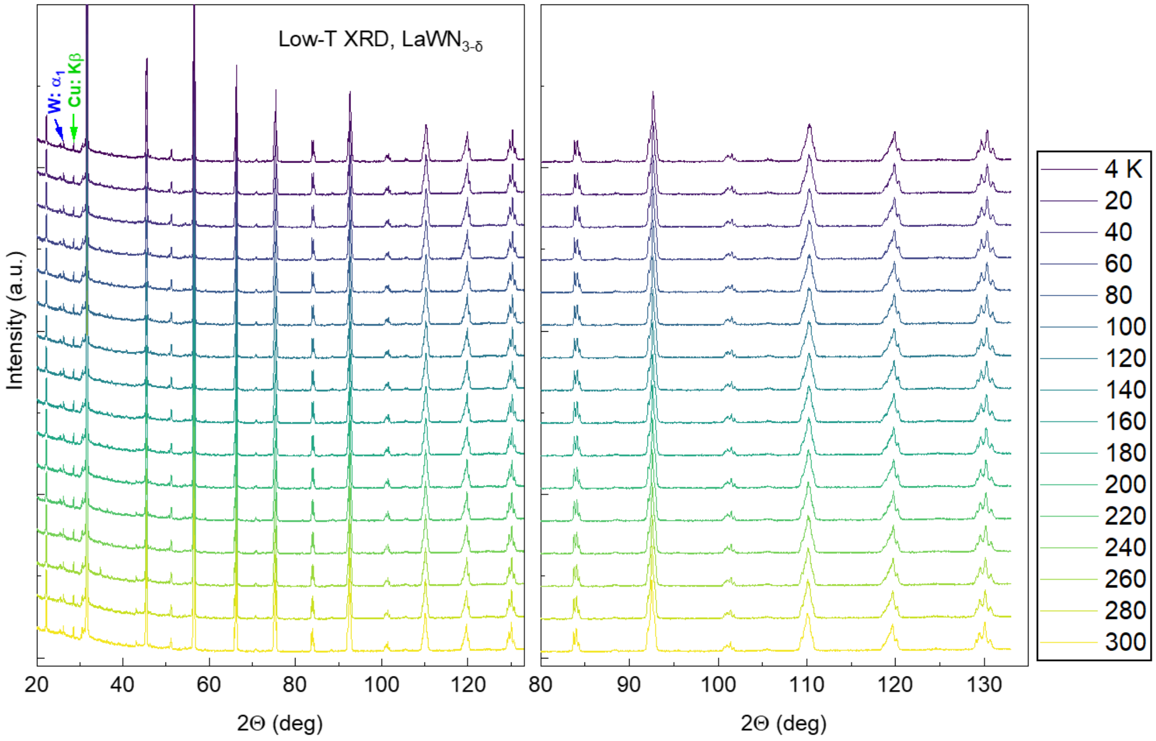

Fig. S19. Low-T XRD patterns taken on cooling. The right panel is an enlargement of the left panel to show the details of peak profiles in the high-2Θ range.

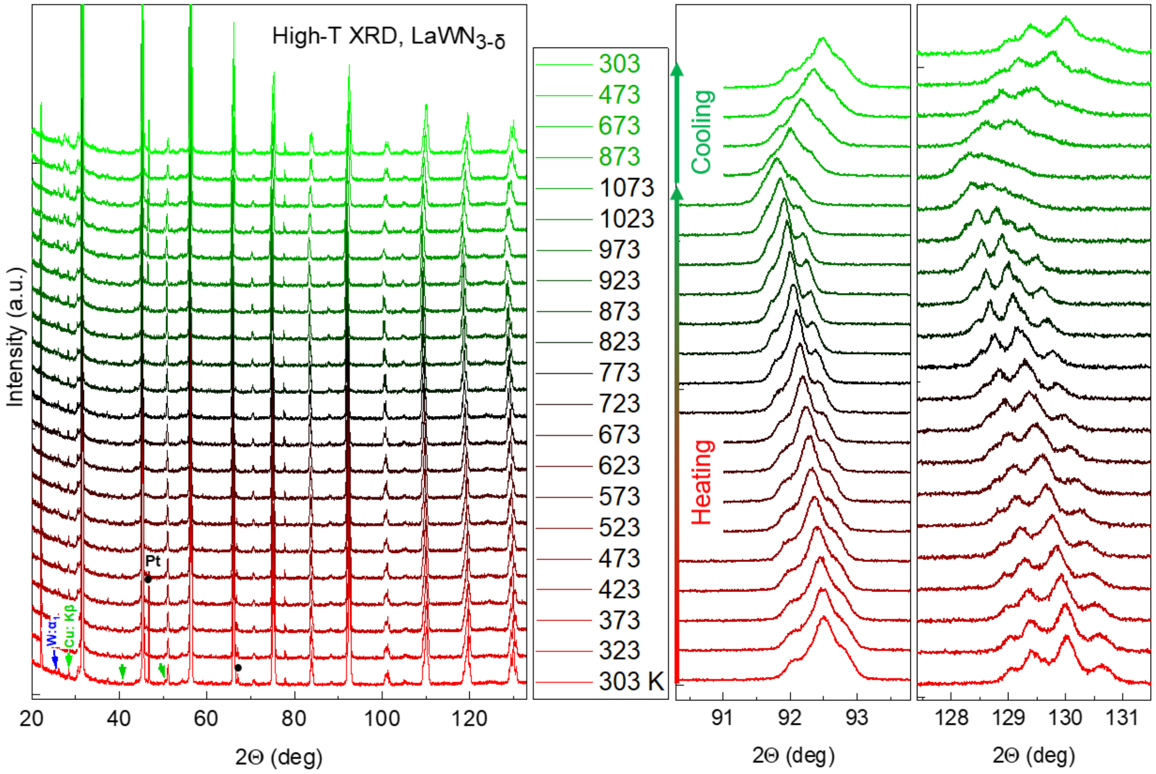

Fig. S20. High-T XRD patterns. The right two panels are enlargements of the left panel to show the details of peak profiles around 2Θ = 92º and 130º.

Variable-T XRD experiments were carried out in the 4 − 1073 K temperature range to study the possible phase transitions and lattice thermal expansion at ambient pressure. To have an accurate control of temperature, at each target temperature the XRD data were taken after holding for 10-15 min to stabilize temperature. The sample position was programmably aligned before data collection.

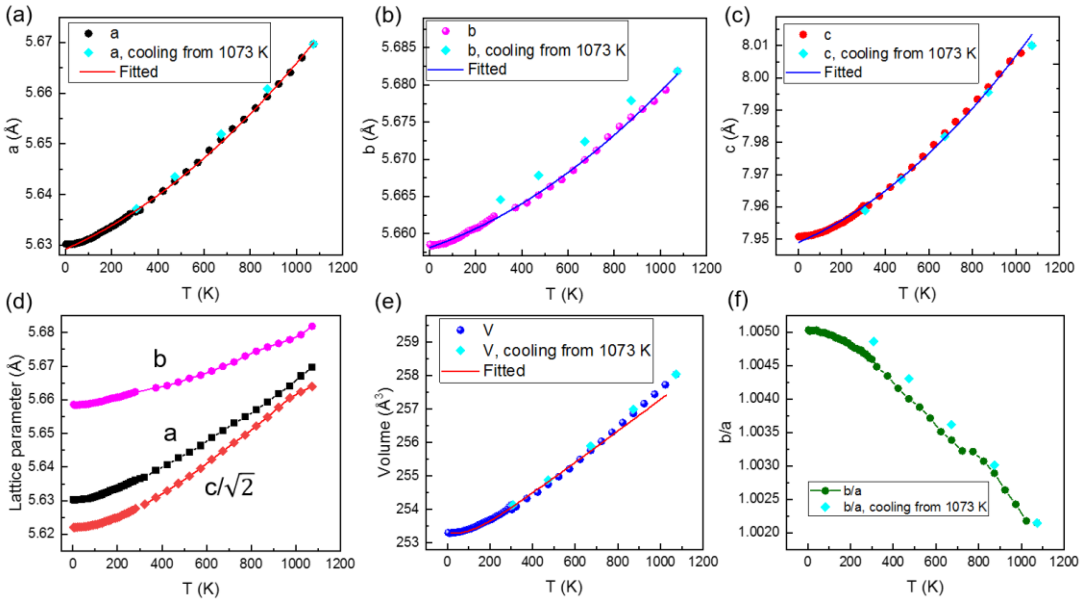

Fig. S21. Refined lattice parameters vs. temperature for *Pna*2$_1$-LaWN$_{3\text{-}\delta}$. (**a**) – (**c**) Plots of *a*, *b*, and *c* *vs.* temperature, respectively. (**d**) Comparison of lattice parameters of *a*, *b*, and *c* in one figure. (**e**) Unit-cell volume vs. temperature. (**f**) Ratio of *b/a* vs. temperature. The axial thermal expansion coefficients are obtained by the fits of lattice parameters *a*, *b*, and *c* vs. temperature data in (a) – (c), respectively, using the equation of $\alpha(T) = \alpha 0 + \alpha_1 T$. Besides, we also use the V-T data in (e) to derive the volume thermal expansion coefficient, using the equation of $V(T) = \mathrm{V}_0 \exp[\int_0^T \alpha(T)\, dT]$. The obtained data are listed in Table S1.

Table S1. Obtained thermal expansion coefficients and Debye temperature (Θ_D).

	α_0 [10^{-6} K^{-1}]	α_1 [10^{-9} K^{-2}]	Θ_D [K]
V	9.01 ± 0.34	1.68 ± 0.06	472
a	3.51 ± 0.10	5.96 ± 0.20	
b	1.87 ± 0.08	3.64 ± 0.16	
c	3.55 ± 0.22	7.40 ± 0.44	

References:

[1] S. D. Kloß, M. L. Weidemann, J. P. Attfield, *Angew. Chem. Int. Ed.* **2021**, 60, 22260.
[2] S. D. Kloß, J. P. Attfield, *Chem. Commun.* **2021**, 57, 10427.
[3] M. Bykov, S. Chariton, H. Fei, et al., *Nat. Commun.* **2019**, 10, 2994.
[4] K. R. Talley, C. L. Perkins, D. R. Diercks, et al., *Science* **2021**, 374, 1488.
[5] C. J. Powell, M. P. Seah, *J. Vac. Sci. Technol., A* **1990**, 8, 735.
[6] J. R. Mycroft, H. W. Nesbitt, A. R. Pratt, *Geochim. Cosmochim. Acta* **1995**, 59, 721.
[7] X. Zhou, C. Gu, G. Song, et al., *Chem. Mater.* **2022**, 34, 9261.
[8] Y.-W. Fang, C. A. J. Fisher, A. Kuwabara, et al., *Phys. Rev. B* **2017**, 95, 014111.
[9] S. Wang, X. Yu, J. Zhang, et al., *Cryst. Growth Des.* **2016**, 16, 351.
[10] F. Y. Xie, L. Gong, X. Liu, et al., *J. Electron. Spectrosc. Relat. Phenom.* **2012**, 185, 112.
[11] M. F. Sunding, K. Hadidi, S. Diplas, et al., *J. Electron. Spectrosc. Relat. Phenom.* **2011**, 184, 399.
[12] K. Y. Koh, S. Zhang, J. Paul Chen, *Chem. Eng. J.* **2020**, 380, 122153.
